# Mutational landscape of marginal zone B-cell lymphomas of various origin: organotypic alterations and diagnostic potential for assignment of organ origin

**DOI:** 10.1007/s00428-021-03186-3

**Published:** 2021-09-08

**Authors:** Visar Vela, Darius Juskevicius, Stefan Dirnhofer, Thomas Menter, Alexandar Tzankov

**Affiliations:** Pathology, Institute of Medical Genetics and Pathology, University Hospital Basel, University of Basel, Schönbeinstrasse 40, 4031 Basel, Switzerland

**Keywords:** Marginal zone lymphoma, Meta-analysis, *FAS*, *KLF2*, NF-κB, *TET2*

## Abstract

This meta-analysis aims to concisely summarize the genetic landscape of splenic, nodal and extranodal marginal zone lymphomas (MZL) in the dura mater, salivary glands, thyroid, ocular adnexa, lung, stomach and skin with respect to somatic variants. A systematic PubMed search for sequencing studies of MZL was executed. All somatic mutations of the organs mentioned above were combined, uniformly annotated, and a dataset containing 25 publications comprising 6016 variants from 1663 patients was created. In splenic MZL, *KLF2* (18%, 103/567) and *NOTCH2* (16%, 118/725) were the most frequently mutated genes. Pulmonary and nodal MZL displayed recurrent mutations in chromatin-modifier-encoding genes, especially *KMT2D* (25%, 13/51, and 20%, 20/98, respectively). In contrast, ocular adnexal, gastric, and dura mater MZL had mutations in genes encoding for NF-κB pathway compounds, in particular *TNFAIP3,* with 39% (113/293), 15% (8/55), and 45% (5/11), respectively. Cutaneous MZL frequently had *FAS* mutations (63%, 24/38), while MZL of the thyroid had a higher prevalence for *TET2* variants (61%, 11/18). Finally, *TBL1XR1* (24%, 14/58) was the most commonly mutated gene in MZL of the salivary glands. Mutations of distinct genes show origin-preferential distribution among nodal and splenic MZL as well as extranodal MZL at/from different anatomic locations. Recognition of such mutational distribution patterns may help assigning MZL origin in difficult cases and possibly pave the way for novel more tailored treatment concepts.

**Supplementary Information:**

The online version contains supplementary material available at 10.1007/s00428-021-03186-3.

## Introduction

Marginal zone lymphomas (MZL) represent 7–8% [1, 2] of all lymphoid neoplasms. The World Health Organization (WHO) [3] subdivides MZL into three distinct entities: splenic MZL (SMZL), nodal MZL (NMZL), and extranodal MZL (EMZL) [2]. The organs most commonly affected by EMZL are the stomach (70%), followed by the lungs (14%), ocular adnexa (12%), thyroid (4%), and the small intestine (1%) [4], while for salivary glands, dura mater, and cutaneous MZL, no incidence data is available [5]. The median age of MZL presentation is 60 years, with a higher proportion of females affected [6]. MZL are mostly indolent with a 5-year overall survival (OS) rate of 85% [6].

There is evidence that some EMZL are associated with and dependent on chronic antigenic stimulation, either by autoantigens or by foreign pathogens, especially bacteria, that lead to accumulation of secondary mucosa-associated lymphoid tissue (MALT) in respective organs due to chronic inflammation, with this MALT serving as soil for neoplastic outgrowth [5]. Infectious agents that have been found to be associated with EMZL are, e.g., *Helicobacter pylori* and *Helicobacter heilmannii* in the stomach, *Achrombacter xylosoxidans* in the lung, *Chlamydophila psittaci* in the ocular adnexa, and *Borrelia burgdorferi* in the skin. Moreover, autoimmune diseases such as Sjögren syndrome and Hashimoto thyroiditis predispose to the development of EMZL [7] (Suppl. Table 1). There is a useful, practical aspect in this consideration: since most EMZL retain their dependence on the respective antigenic stimulation, they may regress upon removal of the antigen, e.g., by antibiotics or by modulation of T-/B-cell interactions by immunomodulatory drugs, even in disseminated disease [8–10].

Compared to other mature small B-cell lymphomas, MZL does not display a disease-defining phenotype. Thus, at occasions, the diagnostic borders among each other, i.e., SMZL, NMZL, and EMZL, as well as within EMZL of various organ origin, and to other small B-cell lymphomas without a defined phenotype are blurred [11, 12].

The pathogenesis of EMZL is linked to several recurrent numerical and structural chromosomal aberrations, i.e., trisomies and chromosomal translocations. Trisomies of chromosomes 3, 12, and 18 are found in 20–30% of EMZL [7]. One of the most common translocations in EMZL, t(11;18)(q21;q21), leads to the fusion of *BIRC3* to *MALT1.* It is tightly linked to EMZL of the lung, and occurs in as much as 45% of cases, followed by the stomach (23%) and the intestine (19%) [7]. Further, this *BIRC3/MALT1* fusion is specific for EMZL, since it is not reported in SMZL or NMZL [7]. On the other hand, partial deletion of the long arm of chromosome 7, del(7)(q31), is found exclusively in SMZL and may even be a biomarker of more aggressive behavior [13, 14]. Another common chromosomal translocation in MZL is t(3;14)(p14;q32) leading to *IGH-FOXP1* rearrangement [7]. Suppl. Table 2 summarizes organotypic chromosomal rearrangements in various MZL.

In the last decade, the genomic landscape of MZL has been extensively studied. With a few exceptions, there seems to be considerable overlap between mutated genes across the various MZL entities and subentities and sites of origin, but this has not yet been integratively analyzed, and being a rare tumor, MZL is still not included in databases such as the International Cancer Genome Consortium (IGGC) and the Cancer Genome Atlas (TGCA).

To address these shortcomings, we performed a meta-analysis of 25 carefully selected PubMed-listed publications reporting on somatic mutations in MZL of various origins, and report here the results of identified variants with consistent and detailed annotation. Whole-genome (WGS), whole exome (WES), targeted high-throughput sequencing (HTS) analysis, and/or Sanger sequencing were read-out methods in these studies.

## Materials and methods

### Literature search

We performed a literature search in October 2020 using PubMed [15] as the primary source. The keywords used and literature research results are detailed in Supplementary Fig. 1. Only studies explicitly stating that cases included had been reviewed and confirmed by staff pathologists were considered.

### Data extraction and annotation

**Fig. 1:**
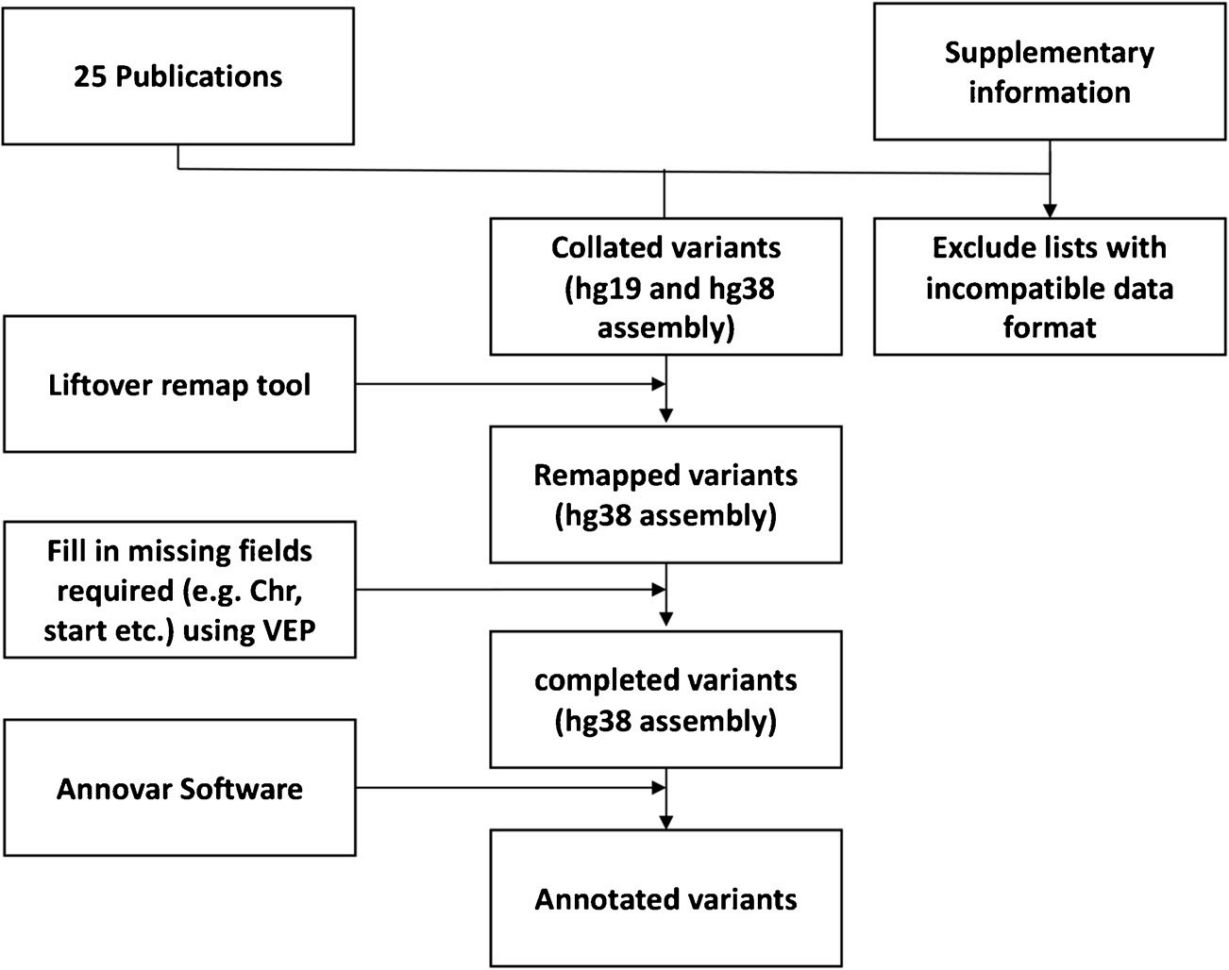
Flowchart explaining the data set compilation and variant assembling strategy implemented in this study

Genomic information was extracted from the supplementary materials of the selected studies and uniformed to the GRCh38-hg38 genome by applying LiftOver - UCSC Genome Browser [15]. The missing information on variants such as genomic location and reference sequence variant effect annotation was obtained with the variant effect predictor (VEP) by Ensemble [15] and Annovar software [16] (Fig. 1).

### Meta-analysis of mutated gene frequencies

The number of mutated and unmutated cases was retrieved and the frequencies of mutations per gene was calculated (Suppl. Table 3). Given the main focus or the current study, namely to assess whether somatic nucleotide variants may be of diagnostic importance, a shortlist was generated for mutated genes with a mutational frequency of > 7.5% in at least one entity (Suppl. Table 4).

Due to format incompatibility and insufficient details, the supplementary list of the study by van den Brand et al. [17] was only used for frequency calculation and not further included. Seven patients from the study of Cascione et al. [18] and 14 from the study of Moody et al. [19] were excluded due to unspecified site of origin.

### Statistical analysis

All statistical calculations were executed with MS Excel or R statistical packages and Statistical Package of Social Sciences (IBM SPSS version 22.0, Chicago, IL, USA) for Windows. Differences of mutational frequencies between EMZL, NMZL, and SMZL entities, as well as between EMZL subentities, were compared using the two-tailed Fisher’s exact test (Suppl. Table 5, Suppl. Fig. 3). The statistical significance threshold was corrected for multiple testing and was set at *p* < 0.017.

## Results

### Filtering of literature, sequencing techniques, and patient characterization

**Fig. 2:**
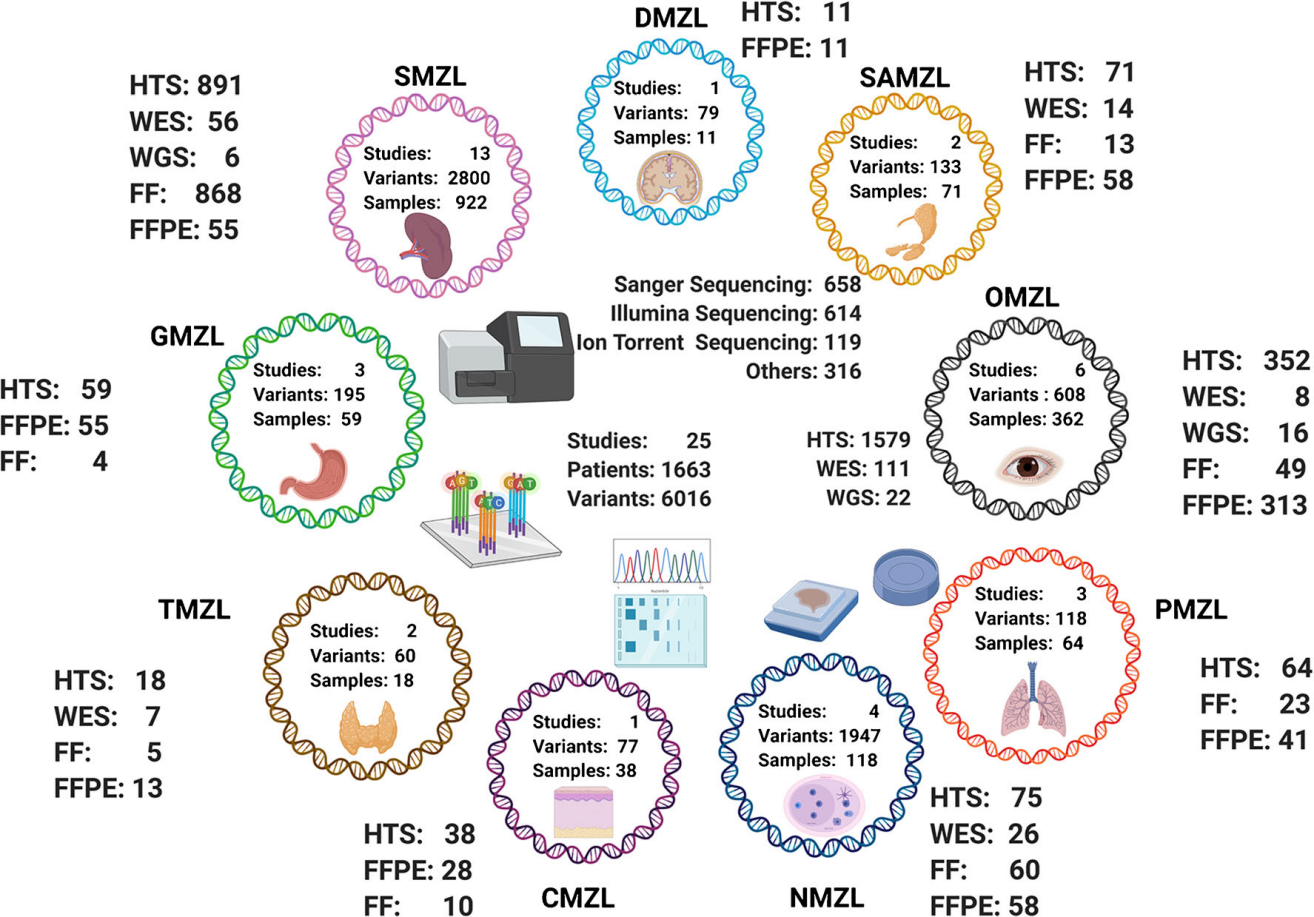
Total number of samples, variants, tissue type material, and sequencing technology applied on 1663 cases for every target organ/site [whole-genome sequencing (WGS) (*n* = 22), whole-exome sequencing (WES) (*n* = 111), Sanger sequencing (*n* = 185), and high-throughput sequencing (HTS) (*n* = 1434)]; twenty-one HTS samples are from an unspecified organ of origin; the different types of tissue source, formalin-fixed paraffin-embedded (FFPE) tissue (*n* = 1327) or fresh frozen (FF) tissue (*n* = 478), are given for each organ/site

After removing duplicate entries, 1602 of 3088 manuscripts were considered unique. After selection based on the criteria detailed above, 142 manuscripts remained for further analysis. Next, all manuscripts and their supplementary data were studied to ensure they reported a full list of variants with appropriate sample information and genetic coordinates. At the end, 25 studies were selected; 3 studies implemented WGS comprising 22 cases, 10 studies applied WES in 111 patients, 2 studies applied Sanger sequencing in 185 probands and 23 studies screened 1434 patients utilizing targeted HTS (Suppl. Table 6); several studies utilized a mix of sequencing strategies. Either formalin-fixed paraffin-embedded (FFPE; *n* = 1327) tissues or/and fresh frozen (FF; *n* = 478) tissues were examined (Fig. 2, Suppl. Table 7).

### Dataset collation and cohort description

**Fig. 3:**
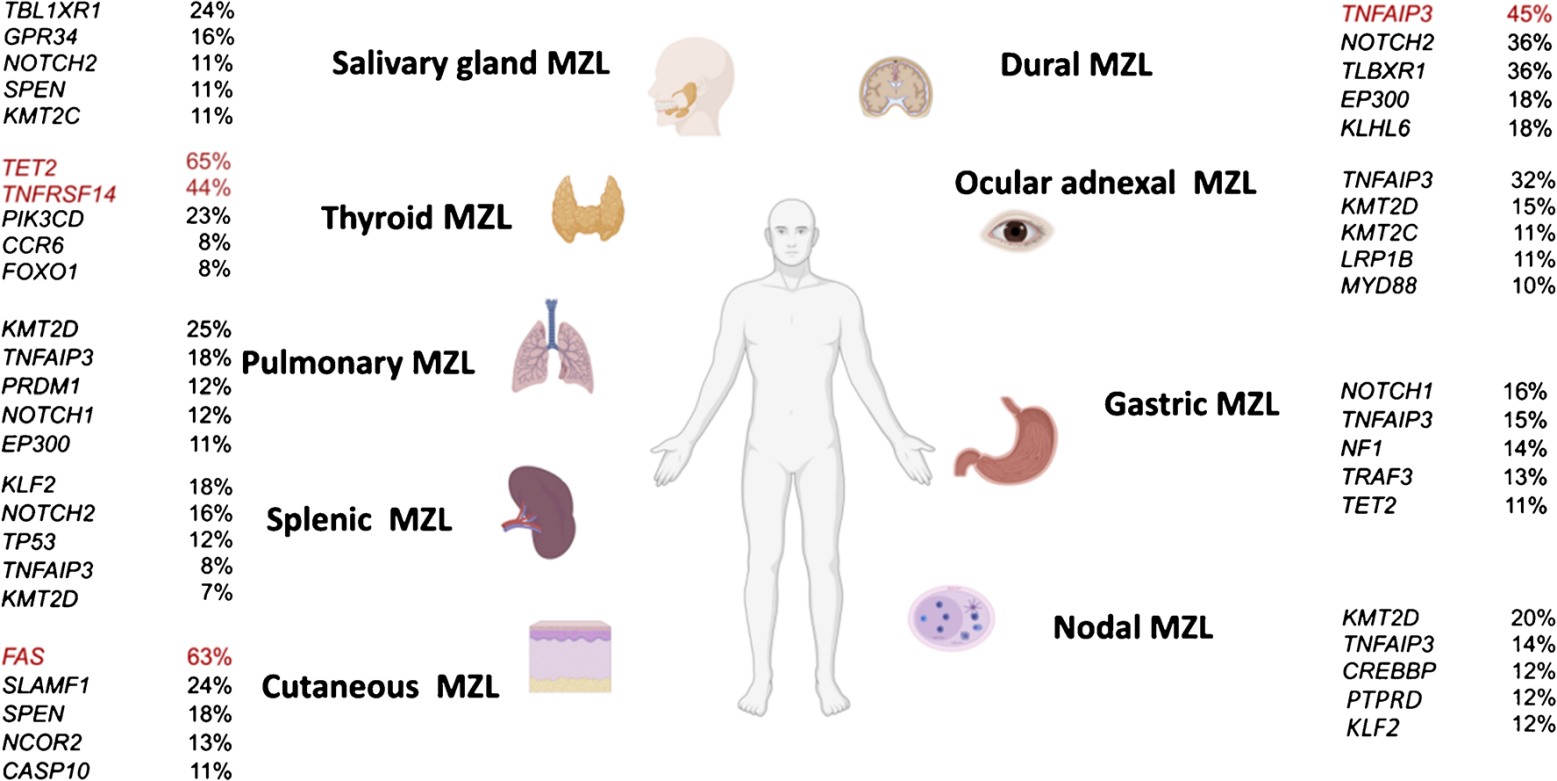
Mutational frequencies of the five most commonly affected genes per entity; genes with frequencies ≥ 40% are highlighted in red

**Fig. 4:**
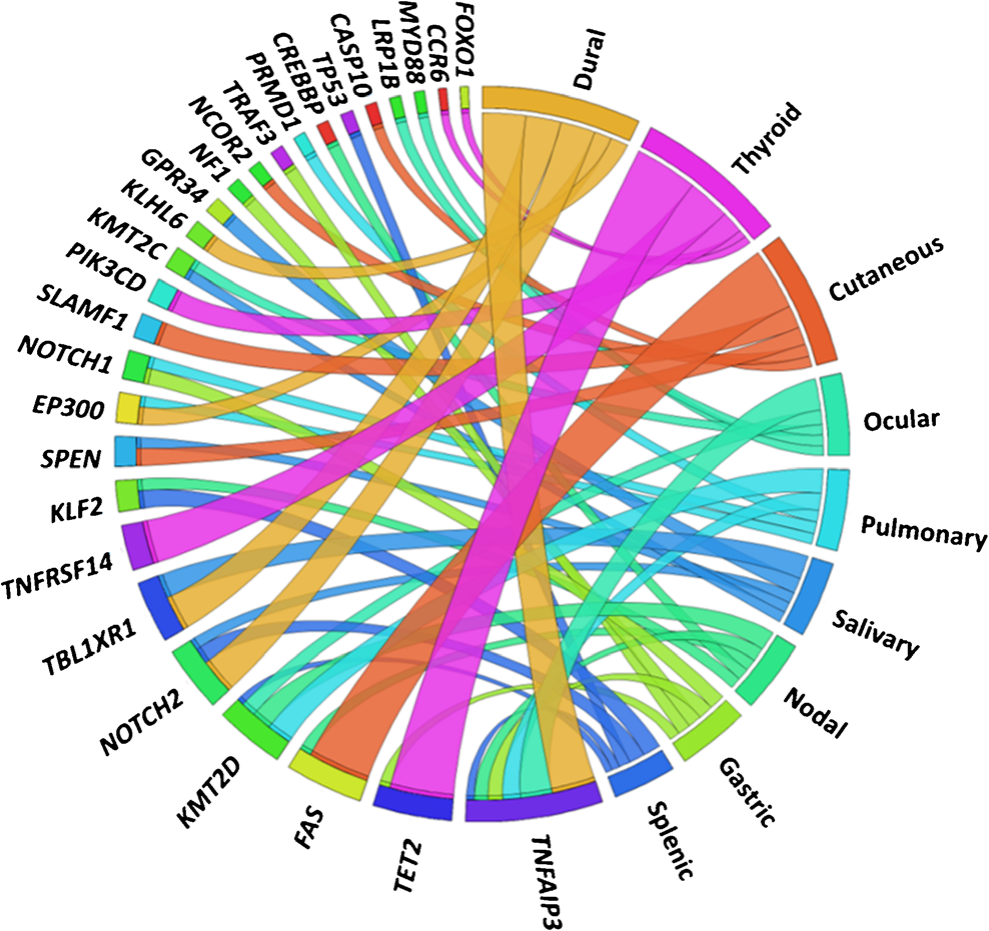
Circos diagram showing the five most frequently mutated genes per entity at various MZL sites; the width of the migration curves indicates the relative frequency of the respective gene mutations

**Fig. 5:**
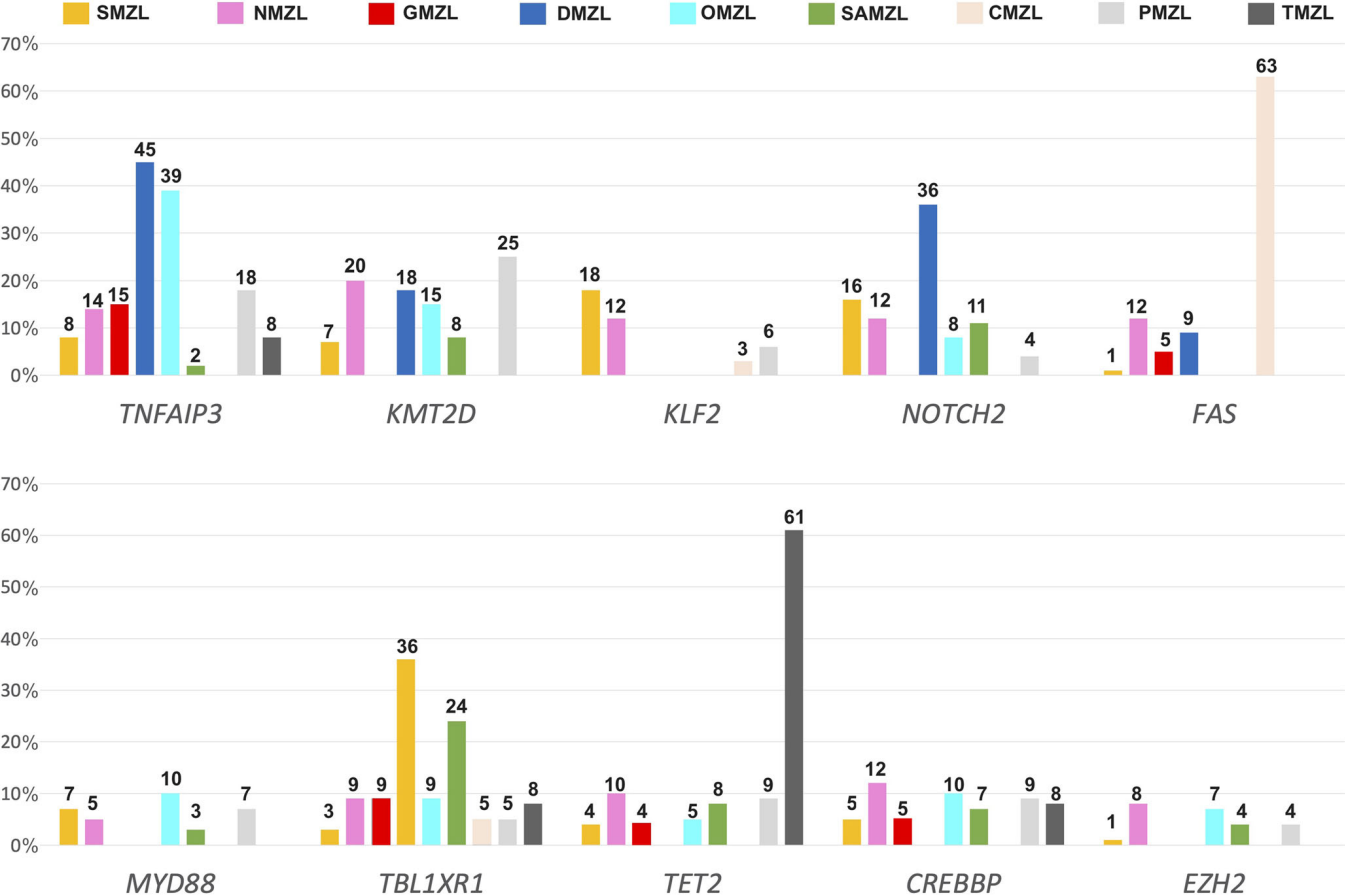
Barplot showing frequencies of ten selected most differentially mutated genes and their distribution throughout the MZL in different anatomic locations; numbers at the top of the bars indicate %

Six thousand sixteen variants in 2553 genes of 1663 cases (Fig. 2) were extracted (Suppl. Table 8). With 13 studies, SMZL was the most comprehensively investigated entity and encompassed 58% of cases in the total cohort, whereas dural (DMZL) and cutaneous MZL (CMZL) accounted for only 3% each, and data was extracted from one publication each per these two respective sites/organs of origin (Fig. 2, Suppl. Fig. 1). Most MZL studies applied NGS-based techniques, only 2 studies on SMZL investigated cases by Sanger sequencing (Suppl. Fig. 2). Table 1 summarizes mutation frequencies per site and per case. Mutations numbers ranged between 1.8 and 27 per case being highest in NMZL. In all entities, single nucleotide variants (SNV) were the most common mutational type. Mutational frequencies in MZL of different entities are represented in Figs. 3, 4, and 5. The statistical comparison results of mutational frequencies by Fisher’s exact test can be found in the Supplementary Table 5.

**Table 1:** Comparative overview of the mutational landscape of different MZL

MZL type	Number of cases	Frequency of cases with mutations	Mean mutations per case*	Mean mutated genes per case*	Types of mutation	Most frequently mutated genes	Most frequently mutated pathways
DMZL	11	100%	7.2	6.5	Missense 72% Frameshift del/ins 10% Nonsense 8% Intronic/Intergenic 6% Splicing site mutation 3% Nonframeshift del/ins 1%	*TNFAIP3* 45% *NOTCH2* 36% *TLBXR1* 36% *EP300* 18% *KLHL6* 18%	Chromatin modifiers 73% NF-κB 63% NOTCH 45%
OMZL	362	67%	2.5	1.78	Missense 59% Nonsense 18% Frameshift del/ins 15% Splicing site mutation 4% Nonframeshift del/ins 2% Intronic/Intergenic 2%	*TNFAIP3* 39% *KMT2D* 15% *CREBBP* 10% *LRP1B* 10% *MYD88* 10%	NF-κB 64% Chromatin modifiers 34% NOTCH 25%
SAMZL	71	70%	2.7	2.3	Missense 67% Nonframeshift del/ins 17% Nonsense 13% Splicing site mutation 2% Frameshift del/ins 1% Intronic/Intergenic 0%	*TBL1XR1* 24% *GPR34* 16% *NOTCH2* 11% *SPEN* 11% *KMT2C* 11%	NOTCH 44% Chromatin modifiers 32% NF-κB 28%
TMZL	18	83%	4	3.1	Missense 60% Frameshift del/ins 15% Nonsense 12% Splicing site mutation 12% Nonframeshift del/ins 1% Intronic/Intergenic 0%	*TET2* 61% *TNFRSF14* 44% *PIK3CD* 23% *SPEN* 17% *CREBBP* 8%	Chromatin modifiers 73% NF-κB 20% NOTCH 20%
PMZL	64	70%	2.6	2.6	Missense 72% Nonsense 16% Frameshift del/ins 8% Nonframeshift del/ins 2% Splicing site mutation 2% Intronic/Intergenic 0%	*KMT2D* 25% *TNFAIP3* 18% *PRDM1* 12% *NOTCH1* 12% *EP300* 11%	Chromatin modifiers 74% NF-κB 42% NOTCH 30%
GMZL	59	64%	5.1	4.4	Missense 74% Frameshift del/ins 12% Nonsense 8% Splicing site mutation 5% Nonframeshift del/ins 1% Intronic/Intergenic 0%	*NOTCH1* 17% *NF1* 16% *TNFAIP3* 15% *TRAF3* 13% *ATM* 13%	NF-κB 61% Chromatin modifiers 55% NOTCH 42%
NMZL	118	75%	29	27	Missense 75% Nonsense 8% Frameshift del/ins 7% Nonframeshift del/ins 5% Splicing site mutation 4% Intronic/Intergenic 1%	*KMT2D* 20% *TNFAIP3* 14% *CREBBP* 12% *FAS* 12% *KLF2* 12%	Chromatin modifiers 70% NOTCH 53% NF-κB 45%
SMZL	922	53%	5.8	5.9	Missense 77% Frameshift del/ins 10% Nonsense 10% Splicing site mutation 2% Nonframeshift del/ins 1% Intronic/Intergenic 0%	*KLF2* 18% *NOTCH2* 16% *TP53* 12% *TNFAIP3* 8% *KMT2D* 7%	NOTCH 53% Chromatin modifiers 43% NF-κB 41%
CMZL	38	84%	2.4	1.8	Missense 70% Splicing site mutation 13% Nonsense 8% Intronic/Intergenic 5% Frameshift del/ins 4% Nonframeshift del/ins 0%	*FAS* 63% *SLAMF1* 24% *SPEN* 18% *NCOR2* 13% *CASP10* 11%	NOTCH 44% NF-κB 6% Chromatin modifiers 6%

*Numbers containing cases both investigated with WGS/WES and targeted sequencing panels

Heat-maps for the distribution of the various mutations per entity/organ/site are provided in Supplementary Figs. 4.1–4.7; for NMZL and SMZL, no heat-maps were constructed due to the large amount of cases and mutations found by WGS and WES, which would have rendered meaningful arrangement confusing.

### Mutational profile of SMZL

Thirteen SMZL studies [20–32] consistently showed that *KLF2* was the most widely mutated gene (18%, 103/567; rather unique for this sub-entity), followed by *NOTCH2* (16%, 118/724) and *TP53* (12%, 59/493) (Figs. 3, 4, and 5). SMZL showed a higher prevalence of *KLF2* and, to a marginal extent, of *NOTCH2* mutations compared to EMZL (4%, 4/90, *p* = 5.73E−04, and 9%, 16/169, *p* = 2.33E−02, respectively). *TP53* was slightly more often mutated in SMZL compared to NMZL (3%, 2/68, *p* = 2.15E−02) and considerably to EMZL (4%, 11/279, *p* = 1.26E−04) (Suppl. Table 5, Suppl. Fig. 3A).

### Mutational profile of NMZL

In four NMZL studies [17, 20, 21, 33], *KMT2D* was reportedly the most frequently mutated gene (20%, 20/98). Genes that were second most commonly mutated, with a frequency of 10%, include *LRP1B* (5/51), *TET2* (5/51), and *TNFRSF14* (10/98). These were followed by *BRAF* (4/51), *EZH2* (4/51), and *HIST1H1E* (8/98), with a frequency of 8% each (Figs. 3, 4, and 5). *KMT2D* was more commonly mutated in NMZL (20%, 20/98) than in SMZL (7%, 28/404, *p* = 1.80E−04). *LRP1B* was more frequently mutated in NMZL (10%, 5/51) compared to SMZL (1%, 4/484, *p* = 6.12E−04). NMZL showed a higher prevalence of *TNFRSF14* mutations (10%, 10/98) compared to SMZL (2%, 6/286, *p* = 1.55E−03). Moreover, we could demonstrate near exclusivity of *BRAF* (8%, 4/51) mutations in NMZL, which reached statistical significance compared to SMZL (1%, 2/301, *p* = 4.74E−03). *EZH2* mutations also appeared more frequently in NMZL (8%, 4/51) than in SMZL (1%, 2/265, *p* = 7.12E−03). Lastly, the *HIST1H1E* mutational rate in NMZL (8%, 8/98) exceeded that in SMZL (2%, 3/188, *p* = 9.35E−03). There was no statistical difference between the mutational profiles of NMZL and EMZL (Suppl. Table 5, Suppl. Fig. 3A).

### Mutational profile of EMZL

Due to the differing cohort numbers, mutational rates are more difficult to describe in EMZL. Ten EMZL [18, 19, 33–40] studies extensively looked for *TNFAIP3* and *TBL1XR1* mutations and detected 140/500 and 66/515 mutant cases, respectively. A total of 29 cases with *NOTCH1* mutations was found in 324 samples, while 14 cases with *KMT2C* mutations were identified in 135 studied instances. Only three studies [33–35] explored *FAS* mutations, which were detected in 26/68 patients. Other genes detected in EMZL studies include *PALB2* (2/11), *JAK3* (11/122), *HIST1H1D* (2/23), and *PTEN* (4/47).

*TNFAIP3* mutations were considerably more detectable in EMZL (28%, 140/500) compared to NMZL (14%, 12/88, *p* = 3.63E−03) and SMZL (8%, 52/628, *p* = 2.21E−18). EMZL displayed a high rate of *TBL1XR1* mutations (13%, 66/515/), which set them apart from SMZL (3%, 7/244, *p* = 4.51E−06). EMZL displayed a high occurrence of *NOTCH1* mutations, differentiating them from SMZL (5%, 24/529, *p* = 1.25E−02). *KMT2C* mutations also appeared to be more frequent in EMZL (10%, 14/135) compared to SMZL (1%, 1/90, *p* = 5.49E−03). *FAS* mutations (38%, 26/68) were more prevalent in EMZL than in SMZL (1%, 4/295, *p* = 2.01E−17) and NMZL (12%, 8/68, *p* = 6.23E−04). *PALB2* mutations were nearly exclusive to EMZL (18%, 2/11), reaching statistical significance compared to SMZL (0.4%, 1/265, *p* = 4.25E−03). *JAK3* was more commonly mutated in EMZL (9%, 11/122) than in SMZL (0.4%, 1/265, *p* = 1.94E−05). *HIST1H1D* mutations were slightly more commonly observable in (9%, 2/23) in EMZL compared to SMZL (1%, 2/265, *p* = 3.32E−02), as were *PTEN* mutations in EMZL (9%, 4/47) compared to SMZL (1%, 1/99, *p* = 3.72E−02) (Suppl. Table 5, Suppl. Fig. 3A).

Comparing mutational frequencies of EMZL occurring in different locations, several important differences could be demonstrated:

Two thyroid MZL (TMZL) studies [18, 19] showed a high prevalence of *TET2* mutations (61%, 11/18), which statistically significantly exceeded that in salivary gland MZL (SAMZL), gastric MZL (GMZL), pulmonary MZL (PMZL), and ocular adnexal MZL (OMZL).

In the two studies with available information on sub-localization of the OMZL (conjunctival versus periorbital) [37, 38], total numbers of mutations in conjunctival OMZL were higher than in periorbital OMZL (median 2 versus 1; mean 2.38 versus 1.56, range 0–9 versus 0–5; *p* = 0.028). *TBL1XR1* mutations were enriched in conjunctival OMZL (8/27 versus 1/17, *p* = 4.63E−02 [37]; 7/22 versus 0/12, *p* = 0.095 [38]).

Compared to other MZL, *FAS* (63%, 24/38) was the most frequently mutated gene in CMZL [35] (Figs. 3, 4, and 5). These characteristic *FAS* mutations were substantially linked to CMZL compared to GMZL and DMZL, displaying such mutations in 5% (1/19, *p* = 3.58E-05) and 9% (1/11, *p* = 1.92E-03) of cases, respectively . Compared to all other MZL, CMZL also showed the highest proportion of splice-site mutations.

A detailed comparison of mutations of EMZL of various sites can be found in the supplementary files.

### Preferred activation of the NOTCH pathway and NF-κB pathway by mutations across different MZL entities

Mutations related to the NOTCH pathway, NF-κB signaling pathway and in genes encoding for chromatin modifiers were grouped and analyzed regarding their role in different MZL. We could observe that mutations related to the NOTCH pathway were rather mutually exclusive to mutations of genes playing a role in the NF-κB pathway and to chromatin modifier-encoding genes. In MZL containing sufficient information density (adequate coverage of genes related to these pathways) to address this issue, 140 cases displayed mutations in both the NF-κB and NOTCH pathway, while 553 cases bore mutations exclusively of genes affecting either pathway, and 242 cases were unmutated, suggesting a nonrandom mutual exclusivity (*p* = 1E−09). Analyzing the different entities separately, statistically significant differences in that consideration were observable in SMZL (*p* = 4E−08) and OMZL (*p* = 8E−03), and as a trend in GMZL. Regarding chromatin modifiers, 207 cases displayed mutual mutations in the NOTCH pathway, while 407 cases bore mutations exclusively of genes affecting either cellular process (*p* = 1E−03). This applied to SMZL (*p* = 1E−03) and OMZL (*p* = 7E−03), and as a trend to SAMZL.

### Concordance between three NMZL WES studies

An additional aim of our study was to perform an unbiased analysis of the genomic landscape of MZL derived from WES as well as targeted HTS to provide an estimation of the overlap of various mutational frequencies of different protein-coding genes. To examine the concordance between studies, we compared WES data of three NMZL studies (Suppl. Fig. 5) [20, 21, 41]. A total of 34 samples sequenced by WES, accounting for 1593 variants, were included in the final list. Similar to a previous report [42] that addressed this concordance in SMZL, our analysis showed a very limited concordance across all three NMZL studies, with only 11 overlapping genes in all three studies.

## Discussion

Our knowledge about the genetic landscape of MZL has increased with the application of new sequencing techniques. However, separate study cohorts, usually derived from archives of one institution, are still limited in size and mutational profiles have been obtained applying different methods. As a result, a general overview of the mutational landscape across all MZL subtypes is lacking. We aimed to perform a comparative meta-analysis of reported genetic variants in various MZL subtypes to address the question of site/organ-of-origin-specific differences.

Some entities displayed similar mutational profiles. These comprise OMZL, PMZL, GMZL, and DMZL, which all showed recurrent *TNFAIP3* mutations and high concordant mutational rates in genes encoding for other compounds of the NF-κB pathway; *TNFAIP3* inhibits NF-κB activation by exerting dual ubiquitin-editing functions [43], thus inactivating mutations of *TNFAIP3* provide an advantage to the cells via activating NF-κB-related signaling.

In contrast, some genes were predominantly mutated in distinct MZL of specific organs/sites, including TMZL that showed a high prevalence of *TET2* mutations and CMZL, which demonstrated a predominance of *FAS* mutations. TET2 is involved in epigenetic regulation; like in *TNFAIP3*, *TET2* mutations are generally loss-of-function mutations that result in an inactive protein and, thus, a net general hypermethylated state of the cells [44]. *TET2* mutations are commonly seen in myeloid neoplasms, ranging from myelodysplastic and overlap syndromes to acute myeloid leukemias as well as in T-cell lymphomas [45]. In B-cell lymphomas in general, they are rather uncommon. Therefore, it is notable that *TET2* mutations occurred in 61% of TMZL, in contrast to all other MZL with *TET2* mutation frequencies < 15% (Fig. 5). Thus, *TET2* mutations can be regarded as rather specific for TMZL and might be of diagnostic help in distinguishing TMZL from other EMZL types of the head and neck.

Another gene primarily mutated in TMZL was *TNFRSF14*. TNFRSF14 is a member of the tumor necrosis factor receptor superfamily and has been described in both follicular lymphomas [46] and diffuse large B-cell lymphomas [47]. It is involved in lymphomagenesis since its inactivating mutations lead to increased B-cell receptor dependent signaling and, via its ligand BTLA, to disrupted interaction of lymphoma B-cells with modulatory T-helper cells [48], thus linking lymphomagenesis to disrupted immune cell crosstalk.

*FAS* was most frequently mutated in CMZL (63%) (Fig. 5), with predominantly splice-site mutations. FAS belongs to the tumor necrosis factor receptor family and its mutations affect the death domain fostering anti-apoptotic properties leading to disrupted protein function and empowering cancer cells with survival advantages [35, 49]. Indeed, Maurus and colleagues reported that all CMZL patients bearing *FAS* mutations showed at least one cutaneous relapse during 84.5 months, while 50% of patients without *FAS* mutations remained free of disease after therapy [35]. *FAS* splice site mutation render cells insensitive to FAS-mediated apoptotic stimuli [50]. *FAS* mutations were, though rarely, also observed in NMZL and SMZL [20, 21, 32]. Thus, *FAS* mutations can be regarded as rather specific for CMZL and might be of diagnostic help in distinguishing primary CMZL from other EMZL types, and pseudolymphoma of the skin.

There were also some other mutations, which tended to be rather organ/site-specific such as *KLF2* and *TP53* in SMZL, *BRAF* and *PTPRD* in NMZL, *NOTCH1* and *NF1* in GMZL, as well as *TBL1XR1* in MZL of the head and neck region. These mutations could also help to provide a tailored diagnostic and may play a role in distinguishing between entities.

In OMZL, the mutational profile of conjunctival and periorbital cases differs, raising the question whether OMZL of different anatomic sub-sites are, e.g., linked to different etiologies and should generally be further subdivided.

Besides single gene comparisons, we also performed analyses of pathways in order to see whether different types of MZL rely on different intracellular signaling conduits. In the majority of cases, we could show that mutations related to the NOTCH pathway were rather mutually exclusive to mutations in the NF-κB pathway and in chromatin modifier-encoding genes, while the two latter showed overlap. This mutual exclusivity was most prominently seen in SMZL and OMZL, and to a lesser extent in SAMZL and GMZL. This again underlines the heterogeneity of MZL and might pave the way towards considerations on tailored targeted treatment approaches for distinct subentities.

The comparably low mutation rates in e.g. GMZL or PMZL might be explained by higher rates of translocations in these entities, which activate the NF-κB pathway. Notably, chromosomal translocations may thus play a more important role in molecular differentiation of MZL entities/subentities than nucleotide-level mutations (Suppl. Table 2). Due to methodological restrictions of the last years, mainly the necessity to perform studies based on FISH, which are both labor- and material-intensive, translocations have not been investigated and compared at large scale between different MZL so far, yet older data suggest certain diagnostic potential linked to distinct rearrangements in MZL [51]. The advent of RNA-based sequencing techniques has the potential to overcome these issues in near future [52].

Limited numbers of patients for some entities/subentities and the heterogeneity of the investigated cohorts without consistent clinical data are potential limitations of the present study, along with differences in sequencing strategies and bioinformatic work-up. Also, the nature of the material employed—either FF or FFPE tissue—may have affected the results. Indeed, discrepancies between the results of single observations, especially when comparing WES-based studies, became obvious, as shown in the Venn diagram for NMZL, which revealed a very small overlap (0.7%) of mutated genes found, although considering the large amount of different genes bearing mutations, this was not surprising (Suppl. Fig. 5). In order to tackle these issues, we homogenized the published data using the algorithms provided and normalized data based on reference genome hg38. Regarding the limitations based on the type of material (FFPE vs FF), Pillonel et al. showed for NMZL an excellent linear correlation between results obtained on either material type as it has been also shown for DLBCL [20, 53], suggesting that at least this might not represent a major confounding factor.

Unfortunately, information regarding infectious agents such as *Helicobacter pylori* (GMZL), *Borrelia burgdorferi* (CMZL), or *Chlamydia psittaci* (OMZL) has not been consistently provided to address the interrelations between mutational profiles and infectious etiology with exception of three studies on OMZL, in which all cases were tested negative for *Chlamydia psittaci*. As the authors of these studies stated in their discussions, infection of OMZL by *Chlamydia psittaci* seems to have a very distinct geographic distribution. Similarly, no information on autoimmune diseases, especially in SAMZL and TMZL, had been provided in the studies included to address mutational differences in instances arising in an autoimmune background.

To conclude, our meta-analysis was able to identify some unique characteristics of organ/site-specific MZL subtypes. *FAS* mutations were found to be restricted to CMZL, while *TET2* and *TNFRSF14* mutations were predominantly found in TMZL. In addition, mutations of *KLF2* and *TP53* (SMZL), *BRAF* and *PTPRD* (NMZL), *NOTCH1* and *NF1* (GMZL), and *TBL1XR1* (MZL of the head and neck region) might help in equivocal instances. Furthermore, *TNFAIP3* mutations and mutations affecting the NF-κB pathway in general are commonly found in OMZL, PMZL, GMZL and DMZL. Recognition of such mutational distribution patterns may be of additional help assigning MZL origin in difficult cases and might possibly pave the way for novel tailored treatment concepts.

## Supplementary Information

ESM 1 (DOCX 18 kb)

ESM 2 (DOCX 16 kb)

Fig. S1 (JPG 892 kb)

Fig. S2 (JPG 162 kb)

Fig. S3 (JPG 965 kb)

Fig. S4.1 (JPG 870 kb)

Fig. S4.2 (JPG 419 kb)

Fig. S4.3 (JPG 2223 kb)

Fig. S4.4 (JPG 796 kb)

Fig. S4.5 (JPG 704 kb)

Fig. S4.6 (JPG 354 kb)

Fig. S4.7 (JPG 321 kb)

Fig. S5 (JPG 332 kb)

Table S1 (XLSX 76 kb)

Table S2 (XLSX 76 kb)

Table S3 (XLSX 45 kb)

Table S4 (XLSX 63 kb)

Table S5 (XLSX 13 kb)

Table S6 (XLSX 58 kb)

Table S7 (XLSX 33 kb)

Table S8 (XLSX 2764 kb)

Table S8.1 (XLSX 3357 kb)

## Data Availability

All raw data is supplied in the supplementary files.

## Declarations

Ethics approval was obtained from the local ethics committee (applicable to the previously published own studies on NMZL, OMZL and PMZL). The procedures used in this study adhere to the tenets of the Declaration of Helsinki.

## References

[1] Joshi M, Sheikh H, Abbi K (2012). Marginal zone lymphoma: old, new, targeted, and epigenetic therapies. Ther Adv Hematol.

[2] Swerdlow S, Campo E, Harris N (2017). WHO classification of tumours of haematopoietic and lymphoid tissues.

[3] Cogliatti S, Bargetzi M, Bertoni F (2016). Supplementum 216: Diagnosis and treatment of marginal zone lymphoma. Swiss Med Wkly.

[4] Khalil MO, Morton LM, Devesa SS (2014). Incidence of marginal zone lymphoma in the United States, 2001–2009 with a focus on primary anatomic site. Br J Haematol.

[5] Troppan K, Wenzl K, Neumeister P, Deutsch A (2015, 2015) Molecular pathogenesis of MALT lymphoma. Gastroenterol Res Pract. 10.1155/2015/102656 10.1155/2015/102656 PMC4397421 25922601

[6] Bertoni F, Coiffier B, Salles G (2011). MALT lymphomas: pathogenesis can drive treatment. Oncology.

[7] Schreuder MI, van den Brand M, Hebeda KM (2017). Novel developments in the pathogenesis and diagnosis of extranodal marginal zone lymphoma. J Hematop.

[8] Kiesewetter B, Raderer M (2020). Immunomodulatory treatment for mucosa-associated lymphoid tissue lymphoma (MALT lymphoma). Hematol Oncol.

[9] Defrancesco I, Arcaini L (2018). Overview on the management of non-gastric MALT lymphomas. Best Pract Res Clin Haematol.

[10] Thieblemont C, Zucca E (2017). Clinical aspects and therapy of gastrointestinal MALT lymphoma. Best Pract Res Clin Haematol.

[11] van den Brand M, van Krieken JHJM (2013). Recognizing nodal marginal zone lymphoma: recent advances and pitfalls. A systematic review. Haematologica.

[12] Pileri S, Ponzoni M (2017). Pathology of nodal marginal zone lymphomas. Best Pract Res Clin Haematol.

[13] Boonstra R, Bosga-Bouwer A, van Imhoff GW (2003). Splenic marginal zone lymphomas presenting with splenomegaly and typical immunophenotype are characterized by allelic loss in 7q31–32. Mod Pathol.

[14] Lloret E, Mollejo M, Mateo MS (1999). Splenic marginal zone lymphoma with increased number of blasts: An aggressive variant?. Hum Pathol.

[15] McLaren W, Gil L, Hunt SE et al (2016) The Ensembl variant effect predictor. Genome Biol 17. 10.1186/s13059-016-0974-4 10.1186/s13059-016-0974-4 PMC4893825 27268795

[16] Wang K, Li M, Hakonarson H (2010). ANNOVAR: functional annotation of genetic variants from high-throughput sequencing data. Nucleic Acids Res.

[17] van den Brand M, Rijntjes J, Hebeda KM (2017). Recurrent mutations in genes involved in nuclear factor-κB signalling in nodal marginal zone lymphoma—diagnostic and therapeutic implications. Histopathology.

[18] Cascione L, Rinaldi A, Bruscaggin A (2019). Novel insights into the genetics and epigenetics of MALT lymphoma unveiled by next generation sequencing analyses. Haematologica.

[19] Moody S, Thompson JS, Chuang S-S (2018). Novel GPR34 and CCR6 mutation and distinct genetic profiles in MALT lymphomas of different sites. Haematologica.

[20] Pillonel V, Juskevicius D, Ng CKY (2018). High-throughput sequencing of nodal marginal zone lymphomas identifies recurrent BRAF mutations. Leukemia.

[21] Spina V, Khiabanian H, Messina M (2016). The genetics of nodal marginal zone lymphoma. Blood.

[22] Clipson A, Wang M, de Leval L (2015). KLF2 mutation is the most frequent somatic change in splenic marginal zone lymphoma and identifies a subset with distinct genotype. Leukemia.

[23] Parry M, Rose-Zerilli MJ, Ljungström V (2015). Genetics and prognostication in splenic marginal zone lymphoma: revelations from deep sequencing. Clin Cancer Res Off J Am Assoc Cancer Res.

[24] Piva R, Deaglio S, Famà R (2015). The Krüppel-like factor 2 transcription factor gene is recurrently mutated in splenic marginal zone lymphoma. Leukemia.

[25] Kiel MJ, Velusamy T, Betz BL (2012). Whole-genome sequencing identifies recurrent somatic NOTCH2 mutations in splenic marginal zone lymphoma. J Exp Med.

[26] Rossi D, Trifonov V, Fangazio M (2012). The coding genome of splenic marginal zone lymphoma: activation of NOTCH2 and other pathways regulating marginal zone development. J Exp Med.

[27] Parry M, Rose-Zerilli MJJ, Gibson J et al (2013) Whole exome sequencing identifies novel recurrently mutated genes in patients with splenic marginal zone lymphoma. PLoS One 8(12):e83244. 10.1371/journal.pone.0083244 10.1371/journal.pone.0083244 PMC3862727 24349473

[28] Campos-Martín Y, Martínez N, Martínez-López A (2017). Clinical and diagnostic relevance of NOTCH2-and KLF2-mutations in splenic marginal zone lymphoma. Haematologica.

[29] Rossi D, Deaglio S, Dominguez-Sola D (2011). Alteration of BIRC3 and multiple other NF-κB pathway genes in splenic marginal zone lymphoma. Blood.

[30] Peveling-Oberhag J, Wolters F, Döring C et al (2015) Whole exome sequencing of microdissected splenic marginal zone lymphoma: a study to discover novel tumor-specific mutations. BMC Cancer 15:773. 10.1186/s12885-015-1766-z 10.1186/s12885-015-1766-z PMC4619476 26498442

[31] Jallades L, Baseggio L, Sujobert P (2017). Exome sequencing identifies recurrent BCOR alterations and the absence of KLF2, TNFAIP3 and MYD88 mutations in splenic diffuse red pulp small B-cell lymphoma. Haematologica.

[32] Martínez N, Almaraz C, Vaqué JP (2014). Whole-exome sequencing in splenic marginal zone lymphoma reveals mutations in genes involved in marginal zone differentiation. Leukemia.

[33] Ganapathi KA, Jobanputra V, Iwamoto F (2016). The genetic landscape of dural marginal zone lymphomas. Oncotarget.

[34] Hyeon J, Lee B, Shin S-H (2018). Targeted deep sequencing of gastric marginal zone lymphoma identified alterations of TRAF3 and TNFAIP3 that were mutually exclusive for MALT1 rearrangement. Mod Pathol.

[35] Maurus K, Appenzeller S, Roth S (2018). Panel sequencing shows recurrent genetic FAS alterations in primary cutaneous marginal zone lymphoma. J Invest Dermatol.

[36] Johansson P, Klein-Hitpass L, Grabellus F (2016). Recurrent mutations in NF-κB pathway components, KMT2D, and NOTCH1/2 in ocular adnexal MALT-type marginal zone lymphomas. Oncotarget.

[37] Jung H, Yoo HY, Lee SH (2017). The mutational landscape of ocular marginal zone lymphoma identifies frequent alterations in TNFAIP3 followed by mutations in TBL1XR1 and CREBBP. Oncotarget.

[38] Vela V, Juskevicius D, Gerlach MM (2020). High throughput sequencing reveals high specificity of TNFAIP3 mutations in ocular adnexal marginal zone B-cell lymphomas. Hematol Oncol.

[39] Johansson P, Klein-Hitpass L, Budeus B et al (2020) Identifying genetic lesions in ocular adnexal extranodal marginal zone lymphomas of the MALT subtype by whole genome, whole exome and targeted sequencing. Cancers (Basel) 12(4):986. 10.3390/cancers12040986 10.3390/cancers12040986 PMC7225979 32316399

[40] Vela V, Juskevicius D, Prince SS (2021). Deciphering the genetic landscape of pulmonary lymphomas. Mod Pathol.

[41] Koh J, Jang I, Choi S et al (2020) Discovery of novel recurrent mutations and clinically meaningful subgroups in nodal marginal zone lymphoma. Cancers 12. 10.3390/cancers12061669 10.3390/cancers12061669 PMC7352856 32585984

[42] Jaramillo Oquendo C, Parker H, Oscier D et al (2019) Systematic review of somatic mutations in splenic marginal zone lymphoma.Sci Rep 9(1):10444. 10.1038/s41598-019-46906-1 10.1038/s41598-019-46906-1 PMC6639539 31320741

[43] Heyninck K, Beyaert R (2005). A20 inhibits NF-κB activation by dual ubiquitin-editing functions. Trends Biochem Sci.

[44] Forbes SA, Beare D, Gunasekaran P (2015). COSMIC: exploring the world’s knowledge of somatic mutations in human cancer. Nucleic Acids Res.

[45] Ko M, Huang Y, Jankowska AM (2010). Impaired hydroxylation of 5-methylcytosine in myeloid cancers with mutant TET2. Nature.

[46] Kotsiou E, Okosun J, Besley C (2016). TNFRSF14 aberrations in follicular lymphoma increase clinically significant allogeneic T-cell responses. Blood.

[47] Lohr JG, Stojanov P, Lawrence MS (2012). Discovery and prioritization of somatic mutations in diffuse large B-cell lymphoma (DLBCL) by whole-exome sequencing. Proc Natl Acad Sci U S A.

[48] Mintz MA, Felce JH, Chou MY (2019). The HVEM-BTLA axis restrains T cell help to germinal center b cells and functions as a cell-extrinsic suppressor in lymphomagenesis. Immunity.

[49] Zhang J, Manley JL (2013). Misregulation of pre-mRNA alternative splicing in cancer. Cancer Discov.

[50] Fisher GH, Rosenberg FJ, Straus SE (1995). Dominant interfering Fas gene mutations impair apoptosis in a human autoimmune lymphoproliferative syndrome. Cell.

[51] Streubel B, Simonitsch-Klupp I, Müllauer L (2004). Variable frequencies of MALT lymphoma-associated genetic aberrations in MALT lymphomas of different sites. Leukemia.

[52] Crotty R, Hu K, Stevenson K (2021). Simultaneous identification of cell of origin, translocations, and hotspot mutations in diffuse large B-cell lymphoma using a single RNA-sequencing assay. Am J Clin Pathol.

[53] Juskevicius D, Lorber T, Gsponer J (2016). Distinct genetic evolution patterns of relapsing diffuse large B-cell lymphoma revealed by genome-wide copy number aberration and targeted sequencing analysis. Leukemia.

