# Supplementary material for: Mutational landscape of marginal zone B-cell lymphomas of various origin: organotypic alterations and diagnostic potential for assignment of organ origin
Source: Virchows Arch. 2021 Sep 8;480(2):403–13. doi: 10.1007/s00428-021-03186-3 (PMC8986713; doi:10.1007/s00428-021-03186-3)
Supplement: Supplementary file 1 — (DOCX 18 kb) [file 428_2021_3186_MOESM1_ESM.docx]

**Supplementary Table Description**

**Supplementary Table 1. Association of extranodal marginal zone lymphoma with various infectious agents and autoimmune disorders.**

**Supplementary Table 2. Association of extranodal marginal zone lymphoma with distribution of characteristic fusion genes.**

**Supplementary Table 3. List of 25 MZL genetic studies containing cases with histopathologically confirmed diagnosis obtained through PubMed search.**

Studies were classified according to the sites/organs of MZL origin.

For Moody et al. 2018, Cascione et al. 2019, Spina et al. 2016, and Pillonel et al. 2018, the respective studies are displayed multiple times as the original publications contain MZL of various sites/organs of origin. The number of cases, mutated cases, unmutated cases, and variant counts from each study are also presented. The number of variants and mutated genes is extracted from the publications.

For Brand et al. 2017, the number of cases are included in the cohort count; however, the number of mutated cases, unmutated cases, and variant counts were left out due to insufficient information given. This study was only used for frequency calculation.

For Koh et al. 2020, the number of cases included in the cohort count is 38; however, the number of mutated cases, and number of variants only includes information from the 8 cases used in WES available in the supplementary files of this study.

**Supplementary Table 4.** Combined raw mutation counts and frequencies of selected genes in investigated entities.

**Supplementary Table 5.** Mutational frequency comparison by Fisher exact test.

**Supplementary Table 6. Total number of cases used for Whole Genome Sequencing, Whole Exome Sequencing, Sanger Sequencing, and High-Throughput/Targeted** **sequencing in every study.**

Information was obtained from the published methodologies. The selected 25 studies have a total number of 1684 cases, Whole Genome Sequencing was applied to 22 patients, Whole Exome Sequencing to 111, Sanger Sequencing to 185, and High-throughput/Targeted Sequencing to 1434.

However, only 1663 cases were used in this study due to the following reasons: 7 cases from the study of Cascione et al. 2019 were excluded due to unspecified site/organ of origin, 14 cases from Moody et al. 2018 were also excluded in this study due to unspecified organ of origin. In addition, for Moody et al. 2018, 179 cases are taken from previous studies and 70 were sequenced for the reported study; data from these studies is considered in this study. The studies Pillonel et al. 2018 and Johansson et al. 2020 applied High-throughput/Targeted Sequencing to all samples.

**Supplementary Table 7.** **Methods and Samples.**

This table outlines the methods used (sequencing techniques), how samples were obtained (tissue origin, matched germline, diagnosis), bioinformatics, data quality, the number of assessed cases, and the total number of cases used in this study. All information was taken from the published methodologies of the different studies found in PubMed.

**Supplementary Table 8. List of all variants obtained from the different studies, filtered, deduplicated, and annotated.**

Sequencing results from the different studies were gathered, filtered, and annotated, resulting in a total of 35 columns. Information collected from publications includes sample/patient IDs, chromosome positions, MZL categories, Ref, Alt, VAF, coverage, and patient counts. All publications were uniformed to the GRCh38 - hg38 genome by applying the LiftOver - UCSC Genome Browser. Total patients count 1663. The missing information of variants such as base-pair location, reference and alternate allele was obtained with the GRCh38 assembly of Variant Effect Predictor (VEP) by Ensemble using the transcript ID and nucleotide change reported.

The final list of variants was annotated using Annovar software to identify the specific variants, exonic variant function, amino acid change, frequency of the variant in specific databases, and scores that predicted how mutations affect protein function. This database does not include information from the study of Brand et al. 2017, due to insufficient data. On the other hand, the database contains incomplete information from Johansson et al. 2020, Moody et al. 2018 (for Thyroid), and Koh et al. 2020. The necessary information was not provided in their supplementaries but was directly taken from their published figures.

**Supplementary Figures Description**

**Suppl. Figure 1.** Flowchart showing the strategy for study selection. The number in squares indicate the number of studies at each step. The studies were divided according to the target organs and duplicates were removed. Those remaining were screened and filtered using inclusion criteria such as methods used, diagnosis, and appropriate sample information. Some studies involved multiple organs of interest and are listed and counted repeatedly in different categories. Thus, the total number of studies at each step does not match with the reported number of unique studies included.

**Suppl. Figure 2.** Pie chart representing the distribution of the different sequencing techniques used in the respective MZL type.

**Suppl. Figure 3A.** Circos diagram showing the frequently mutated genes from the fisher test in correspondence to the various EMZL, NMZL and SMZL entities. The width of the migration curves indicates the mutational frequency.

**Suppl. Figure 3B.** Circos diagram showing the frequently mutated genes from the fisher test only within the EMZL entity. The width of the migration curves indicates the mutational frequency.

**Suppl. Figures 4.1-4.7:** Heat map plots of the somatic variants in different MZL subentities. Only mutated cases of each entity are included.

**Suppl. Figure 4.1.** Heat map plot of the somatic variants in different studies of gastric MZL showing all nonsynonymous mutations detected by targeted high throughput sequencing (n=59). Each column represents a primary tumor; each row represents a gene ordered top-down in decreasing order of detection frequency; when multiple mutations are present in the same gene, only one mutation is displayed; these apply to all following heatmaps.

**Suppl. Figure 4.2.** Heat map plot of the somatic variants in different studies of dural MZL showing all nonsynonymous mutations detected by targeted high throughput sequencing (n=11).

**Suppl. Figure 4.3.** Heat map plot of the somatic variants in different studies of ocular MZL showing all nonsynonymous mutations detected by targeted high throughput sequencing (n=352), whole genome sequencing (n=16), and whole exome sequencing (n=8). This heatmap includes only mutated cases (n=242).

**Suppl. Figure 4.4.** Heat map plot of the somatic variants in different studies of pulmonary MZL showing all nonsynonymous mutations detected by targeted high throughput sequencing (n=64). This heatmap only includes mutated cases (n=45).

**Suppl. Figure 4.5** Heat map plot of the somatic variants in different studies of salivary gland MZL showing all nonsynonymous mutations detected by targeted high throughput sequencing (n=71), and whole exome sequencing (n=14). This heatmap only includes mutated cases (n=50).

**Suppl. Figure 4.6.** Heat map plot of the somatic variants in different studies of thyroid MZL showing all nonsynonymous mutations detected by targeted high throughput sequencing (n=18), and whole exome sequencing (n=7). This heatmap only includes mutated cases (n=15).

**Suppl. Figure 4.7.** Heat map plot of the somatic variants in different studies of cutaneous MZL showing all nonsynonymous mutations detected by targeted high throughput sequencing (n=38). This heatmap only includes mutated cases (n=32).

**Suppl. Figure 5.** Venn diagram showing the number of gene overlaps in three WES studies of nodal MZL.
