# Supplementary material for: Mutational landscape of marginal zone B-cell lymphomas of various origin: organotypic alterations and diagnostic potential for assignment of organ origin
Source: Virchows Arch. 2021 Sep 8;480(2):403–13. doi: 10.1007/s00428-021-03186-3 (PMC8986713; doi:10.1007/s00428-021-03186-3)
Supplement: Supplementary file 2 — (DOCX 16 kb) [file 428_2021_3186_MOESM2_ESM.docx]

**Supplementary file: detailed description and comparisons of mutations in EMZL**

**Mutations in EMZL in different anatomic locations**

***Dural MZL***

In the single DMZL study [[1]](https://www.zotero.org/google-docs/?L8iVb9), *TNFAIP3* (45%, 5/11) was the most commonly mutated gene, followed by *NOTCH2* (36%, 4/11), and *TBL1XR1* (36%, 4/11) (Figure 3/4/5). A statistically significant higher prevalence of *TNFAIP3* mutations in DMZL (45%, 5/11) compared to MZL of the salivary glands (SAMZL) (2%, 1/58, p=2.27E-04) and, subsignificantly, to gastric (GMZL) (15%, 8/55, p=3.26E-02) was noted. *TBL1XR1* was slightly more commonly mutated in DMZL (36%, 4/11) compared to ocular adnexal (OMZL) (9%, 27/289, p=1.85E-02), pulmonary (PMZL) (5%, 2/41, p=1.42E-02), GMZL (9%, 5/55, p=3.56E-02), and cutaneous (CMZL) (5%, 2/36, p=1.79E-02). In addition, *NOTCH2* appeared more frequently in DMZL (36%, 4/11) than in OMZL (8%, 8/106, p=1.46E-02), and, subsignificantly, in PMZL (4%, 1/23, p=2.89E-02) (Suppl. Table 5, Suppl. Figure 3B).

***Ocular adnexal MZL***

Six OMZL studies [[2–7]](https://www.zotero.org/google-docs/?R4pCXE) highlighted a high frequency rate of *TNFAIP3* mutations (39%, 113/293), followed by mutations of *KMT2D* (15%, 26/178) (Figure 3/4/5). Yet, there was no significant difference in these mutational frequencies to other EMZL (Suppl. Table 5, Suppl. Figure 3B).

In the two studies with available information on sub-localization of the OMZL (conjunctival versus periorbital) [[5, 6]](https://www.zotero.org/google-docs/?yYlflv), total numbers of mutations in conjunctival OMZL were more higher than in periorbital OMZL (median 2 versus 1; mean 2.38 versus 1.56, range 0-9 versus 0-5; p=0.028). *TBL1XR1* mutations were enriched in conjunctival OMZL (8/27 versus 1/17, p=4.63E-02 [[5]](https://www.zotero.org/google-docs/?wMooYX)); 7/22 versus 0/12, p=0.095 [[6]](https://www.zotero.org/google-docs/?j2tghd)).

***MZL of the salivary glands***

Two studies on SAMZL [[2, 3]](https://www.zotero.org/google-docs/?yQwc78) showed *TBL1XR1* (24%, 14/58) to be the most frequently mutated gene, followed by *GPR34* (16%, 9/58) (Figure 3/4/5). There was no significant difference in these mutational frequencies to other EMZL (Suppl. Table 5, Suppl. Figure 3B).

***Thyroid MZL***

Two thyroid MZL (TMZL) studies [[2, 3]](https://www.zotero.org/google-docs/?VK0oD9) showed high prevalence of *TET2* mutations (61%, 11/18), followed by *TNFRSF14* (44%, 8/18) and *PIK3CD* (23%, 3/13) (Figure 3/4/5). The high mutational rate of *TET2* in TMZL significantly exceeded that in SAMZL (8%, 6/71, p=6.34E-06), GMZL (4%, 2/55, p=5.60E-07), PMZL (9%, 6/64, p=1.56E-05) and OMZL (5%, 9/192, p=5.89E-09). The frequency of *TNFRSF14* mutations in TMZL (44%, 8/18) exceeded that in GMZL (8%, 3/36, p=3.60E-03), SAMZL (3%, 2/58, p=7.88E-05), PMZL (5%, 2/41, p=6.04E-04), and OMZL (4%, 8/192, p=4.77E-06). In addition, *PIK3CD* mutations in TMZL (23%, 3/13) were more prevalent than in OMZL (2%, 3/138, p= 8.70E-03) (Suppl. Table 5, Suppl. Figure 3B).

***Pulmonary MZL***

In all three studies on PMZL [[2, 3, 8]](https://www.zotero.org/google-docs/?q0PpG1), *KMT2D* was reported as the most commonly mutated gene with a frequency of 25% (13/51), followed by *PRDM1* (12%, 6/51) (Figure 3/4). Interestingly, *KMT2D* (25%, 13/51) mutations in PMZL were slightly more prevalent than in SAMZL (8%, 6/71, p=2.08E-02). Additionally, *PRDM1* mutation occurrence (12%, 6/51) in PMZL subsignificantly exceeded that in OMZL (4%, 7/192, p=3.33E-02) (Suppl. Table 5, Suppl. Figure 3B).

***Gastric MZL***

Three studies on GMZL [[2, 3, 9]](https://www.zotero.org/google-docs/?zMF7pa) reported frequent mutations of *NOTCH1* and *TRAF3*, accounting for 17% (10/59) and 13% (7/55), respectively (Figure 3/4). These more commonly observable mutations of *NOTCH1* and *TRAF3* in GMZL, were slightly to significantly more rarely observable in OMZL (6%, 9/144, p=3.05E-02, and 2%, 3/138, p=6.37E-03, respectively) (Suppl. Table 5, Suppl. Figure 3B).

***Cutaneous MZL***

Compared to other MZL, *FAS* (63%, 24/38) was the most frequently mutated gene in CMZL [[10]](https://www.zotero.org/google-docs/?erNEsF) (Figure 3/4/5). These characteristic *FAS* mutations (63%, 24/38) were considerably linked to CMZL compared to GMZL (5%, 1/19, p=3.58E-05) and DMZL (9%, 1/11, p=1.92E-03) (Suppl. Table 5, Suppl. Figure 3B).

**Mutational types and significant differences in the distribution of recurrently mutated genes in MZL of various sites/organs**

72% (57/79) of all variants in DMZL were missense mutations. Mutations in NMZL, SMZL, and GMZL were most often missense as well (75%, 1470/1946, 77%, 2167/2800, and 74%, 144/195, respectively).

OMZL showed the highest prevalence of nonsense mutations with a proportion of 18% (108/608), these mainly occurred in *TNFAIP3, KMT2D* and surprisingly in *MYD88*, which accounted for 61% (66/108) of all nonsense mutations. Interestingly, while mutations in PMZL were mostly missense, 72% (85/118), PMZL also displayed similar frequencies of nonsense mutations like OMZL 16% (19/118).

SAMZL showed, in comparison to all other MZL, the highest proportion of non-frameshift insertions/deletions, 17% (22/133) (Table 1). This was mainly because all three commonly mutant genes in SAMZL, *NOTCH1,* *TBL1XR1* and *TRAF3,* displayed together 64% (14/22) of all nonframeshift deletions/insertions.

Compared to all other MZL, CMZL showed the highest proportion of splice-site mutations, 13% (10/77), mainly because of high frequency of splice-site *FAS* of 38% (9/24). In TMZL, frameshift insertions/deletions were with 15% (9/60) most commonly observable compared to all other MZL entities and subentities. This is explained because of the high prevalence of *TET2* mutations in this entity with 38% (8/21) being frameshift insertions/deletions.

**References:**

[1. Ganapathi KA, Jobanputra V, Iwamoto F, et al (2016) The genetic landscape of dural marginal zone lymphomas. Oncotarget 7:43052–43061. https://doi.org/10.18632/oncotarget.9678](https://www.zotero.org/google-docs/?S0yO7q)

[2. Cascione L, Rinaldi A, Bruscaggin A, et al (2019) Novel insights into the genetics and epigenetics of MALT lymphoma unveiled by next generation sequencing analyses. Haematologica 104:e558–e561. https://doi.org/10.3324/haematol.2018.214957](https://www.zotero.org/google-docs/?S0yO7q)

[3. Moody S, Thompson JS, Chuang S-S, et al (2018) Novel GPR34 and CCR6 mutation and distinct genetic profiles in MALT lymphomas of different sites. Haematologica 103:1329–1336. https://doi.org/10.3324/haematol.2018.191601](https://www.zotero.org/google-docs/?S0yO7q)

[4. Johansson P, Klein-Hitpass L, Grabellus F, et al (2016) Recurrent mutations in NF-κB pathway components, KMT2D, and NOTCH1/2 in ocular adnexal MALT-type marginal zone lymphomas. Oncotarget 7:62627–62639. https://doi.org/10.18632/oncotarget.11548](https://www.zotero.org/google-docs/?S0yO7q)

[5. Jung H, Yoo HY, Lee SH, et al (2017) The mutational landscape of ocular marginal zone lymphoma identifies frequent alterations in TNFAIP3 followed by mutations in TBL1XR1 and CREBBP. Oncotarget 8:17038–17049. https://doi.org/10.18632/oncotarget.14928](https://www.zotero.org/google-docs/?S0yO7q)

[6. Vela V, Juskevicius D, Gerlach MM, et al (2020) High throughput sequencing reveals high specificity of TNFAIP3 mutations in ocular adnexal marginal zone B-cell lymphomas. Hematol Oncol 38:284–292. https://doi.org/10.1002/hon.2718](https://www.zotero.org/google-docs/?S0yO7q)

[7. Johansson P, Klein-Hitpass L, Budeus B, et al (2020) Identifying Genetic Lesions in Ocular Adnexal Extranodal Marginal Zone Lymphomas of the MALT Subtype by Whole Genome, Whole Exome and Targeted Sequencing. Cancers 12:. https://doi.org/10.3390/cancers12040986](https://www.zotero.org/google-docs/?S0yO7q)

[8. Vela V, Juskevicius D, Prince SS, et al (2021) Deciphering the genetic landscape of pulmonary lymphomas. Mod Pathol 34:371–379. https://doi.org/10.1038/s41379-020-00660-2](https://www.zotero.org/google-docs/?S0yO7q)

[9. Hyeon J, Lee B, Shin S-H, et al (2018) Targeted deep sequencing of gastric marginal zone lymphoma identified alterations of TRAF3 and TNFAIP3 that were mutually exclusive for MALT1 rearrangement. Mod Pathol 31:1418–1428. https://doi.org/10.1038/s41379-018-0064-0](https://www.zotero.org/google-docs/?S0yO7q)

[10. Maurus K, Appenzeller S, Roth S, et al (2018) Panel Sequencing Shows Recurrent Genetic FAS Alterations in Primary Cutaneous Marginal Zone Lymphoma. J Invest Dermatol 138:1573–1581. https://doi.org/10.1016/j.jid.2018.02.015](https://www.zotero.org/google-docs/?S0yO7q)
