# Supplementary figures and images for: Mutational landscape of marginal zone B-cell lymphomas of various origin: organotypic alterations and diagnostic potential for assignment of organ origin
Source: Virchows Arch. 2021 Sep 8;480(2):403–13. doi: 10.1007/s00428-021-03186-3 (PMC8986713; doi:10.1007/s00428-021-03186-3)

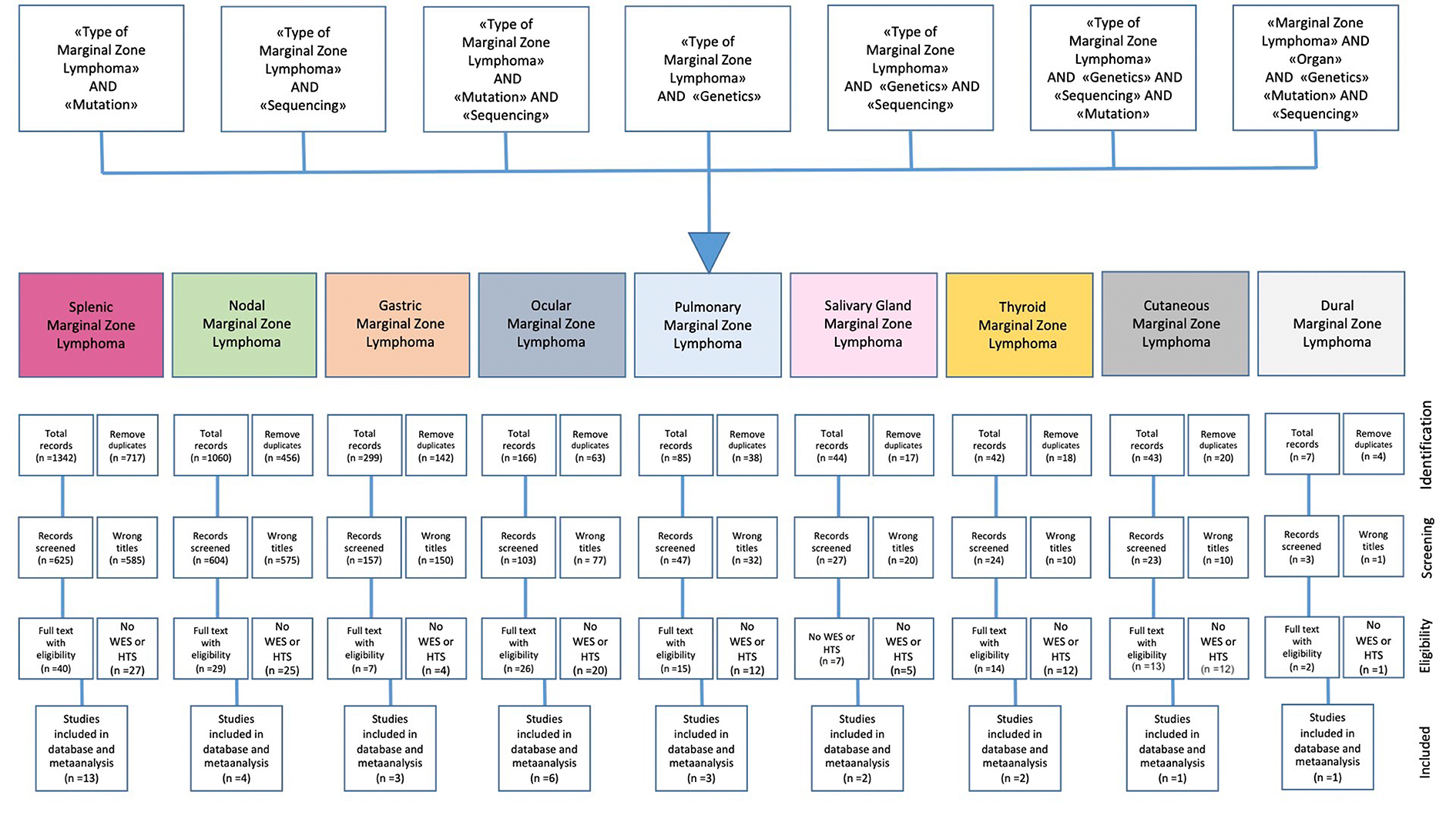

Supplement: Supplementary file 3 — (JPG 892 kb) [file 428_2021_3186_MOESM3_ESM.jpg]

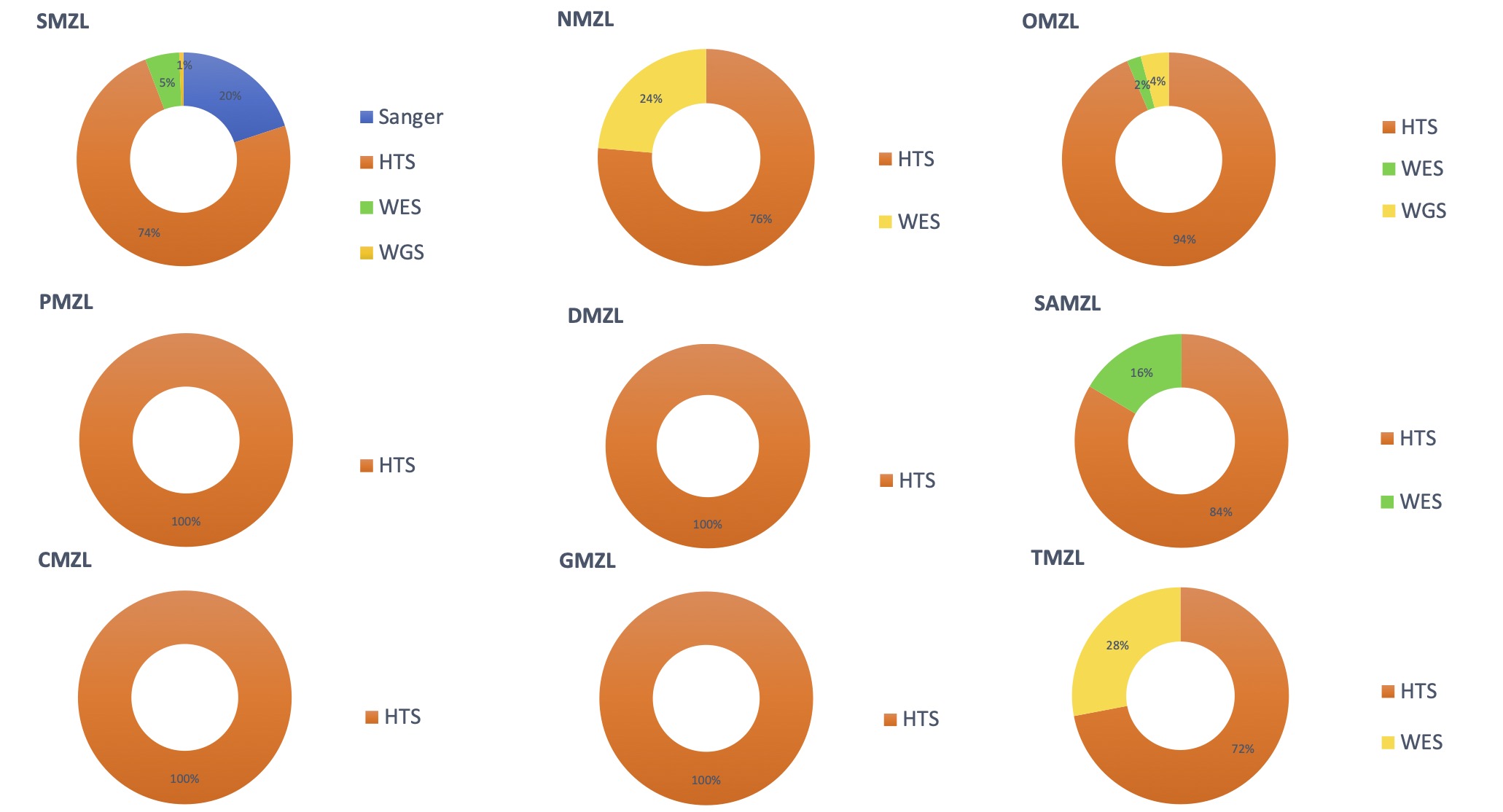

Supplement: Supplementary file 4 — (JPG 162 kb) [file 428_2021_3186_MOESM4_ESM.jpg]

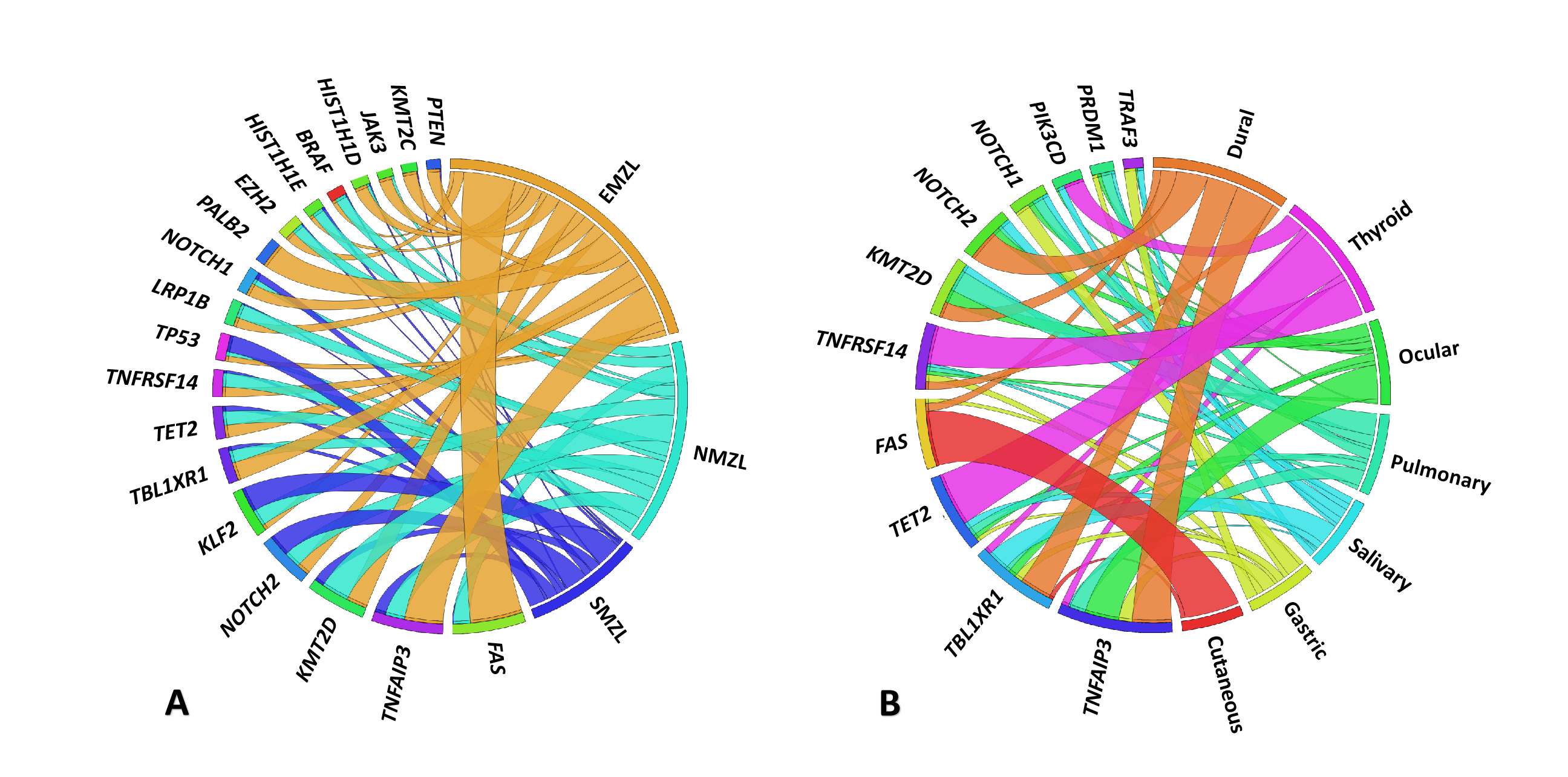

Supplement: Supplementary file 5 — (JPG 965 kb) [file 428_2021_3186_MOESM5_ESM.jpg]

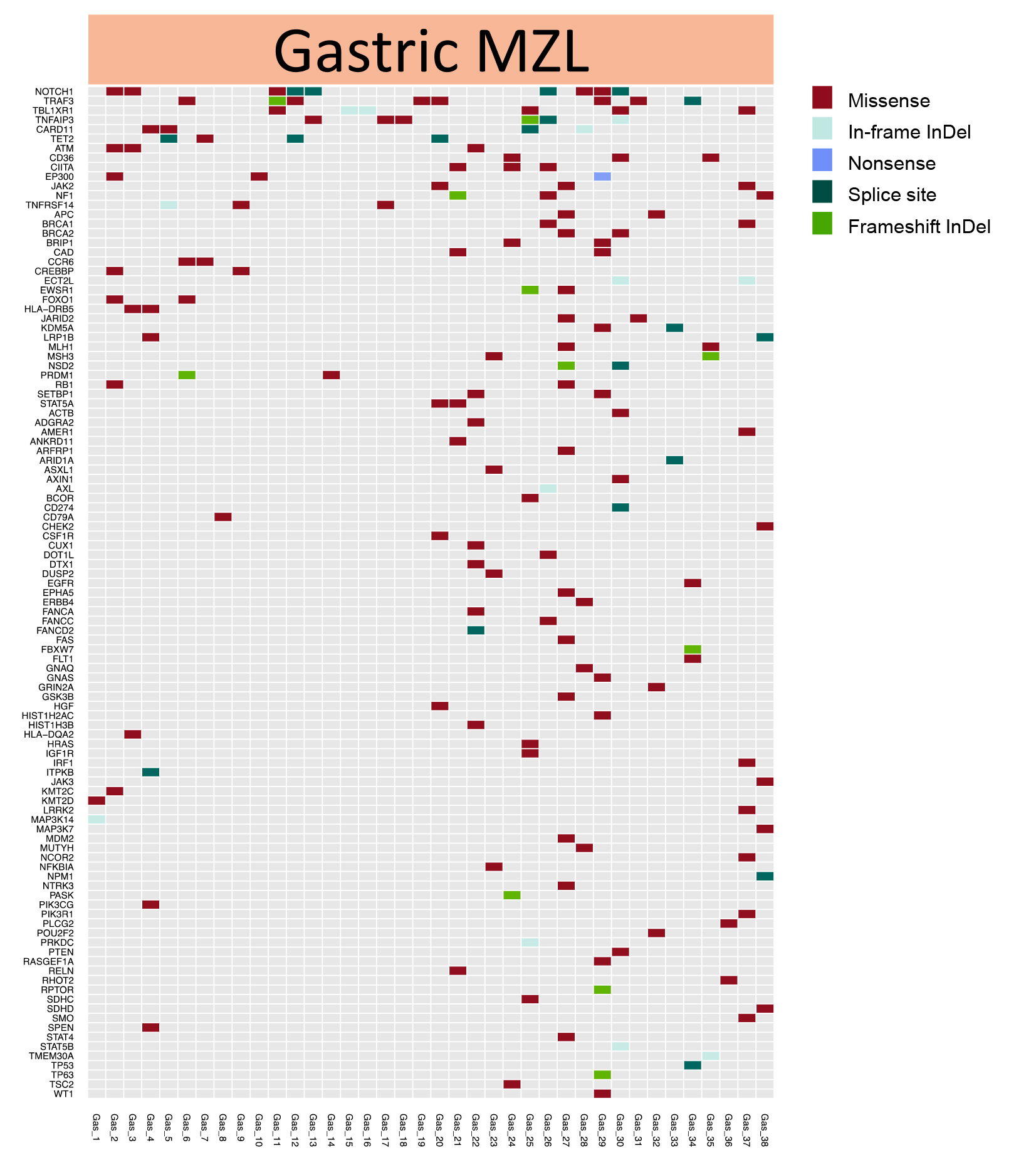

Supplement: Supplementary file 6 — (JPG 870 kb) [file 428_2021_3186_MOESM6_ESM.jpg]

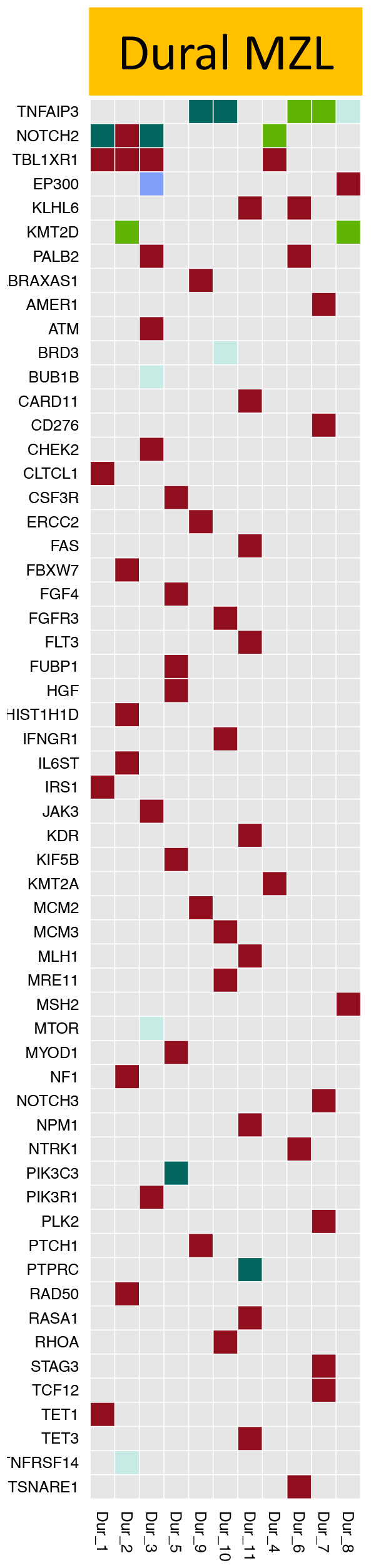

Supplement: Supplementary file 7 — (JPG 419 kb) [file 428_2021_3186_MOESM7_ESM.jpg]

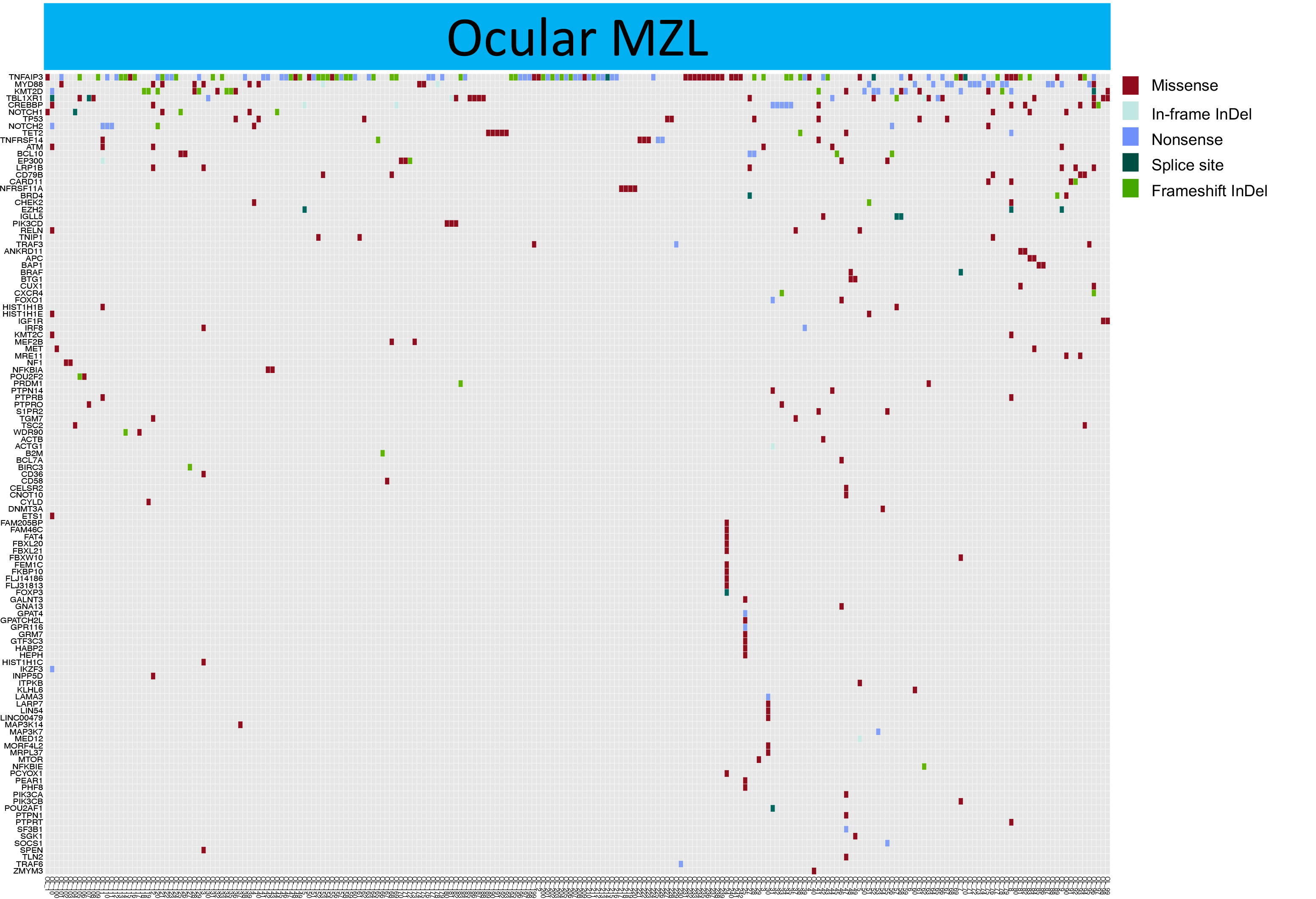

Supplement: Supplementary file 8 — (JPG 2223 kb) [file 428_2021_3186_MOESM8_ESM.jpg]

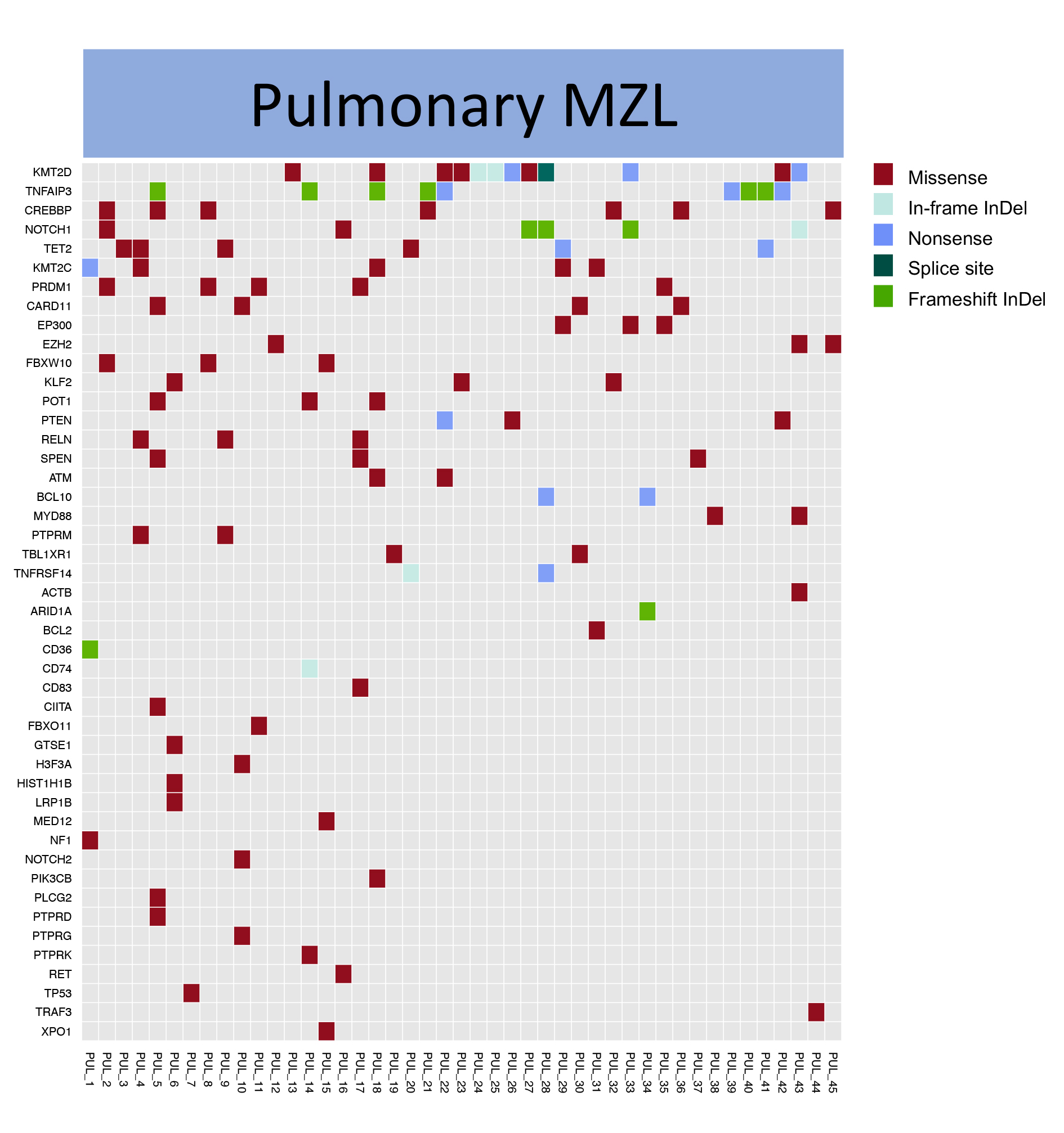

Supplement: Supplementary file 9 — (JPG 796 kb) [file 428_2021_3186_MOESM9_ESM.jpg]

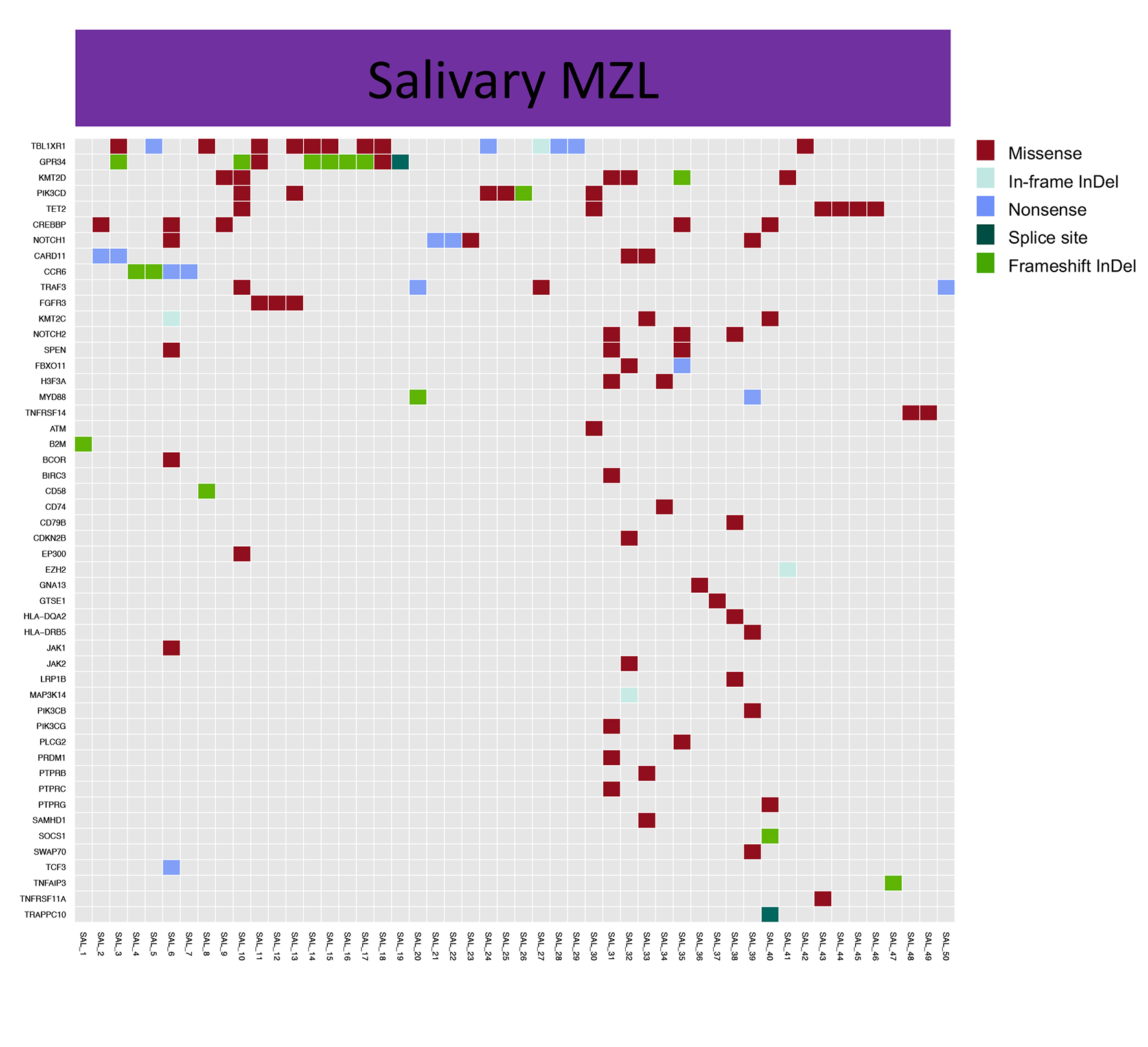

Supplement: Supplementary file 10 — (JPG 704 kb) [file 428_2021_3186_MOESM10_ESM.jpg]

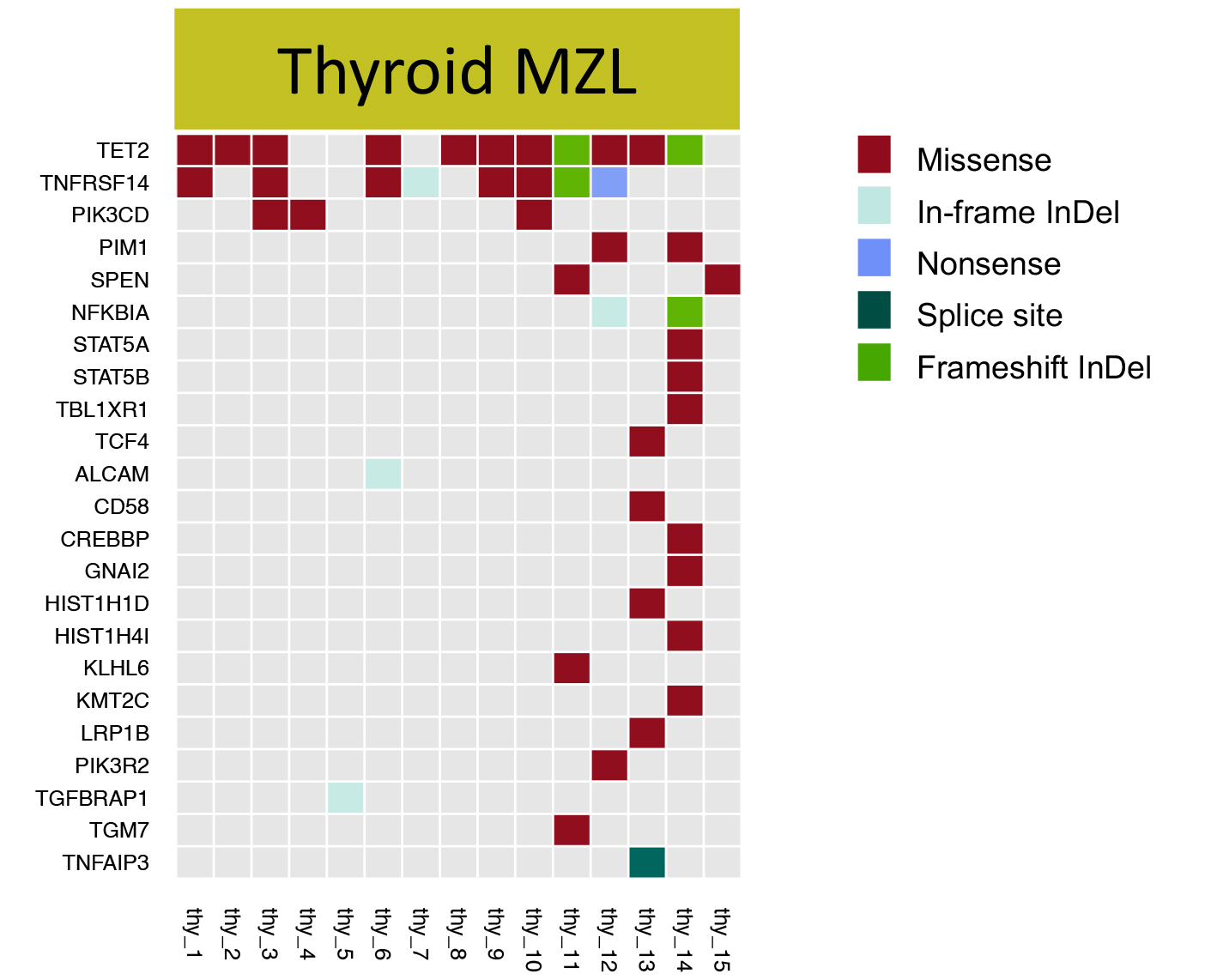

Supplement: Supplementary file 11 — (JPG 354 kb) [file 428_2021_3186_MOESM11_ESM.jpg]

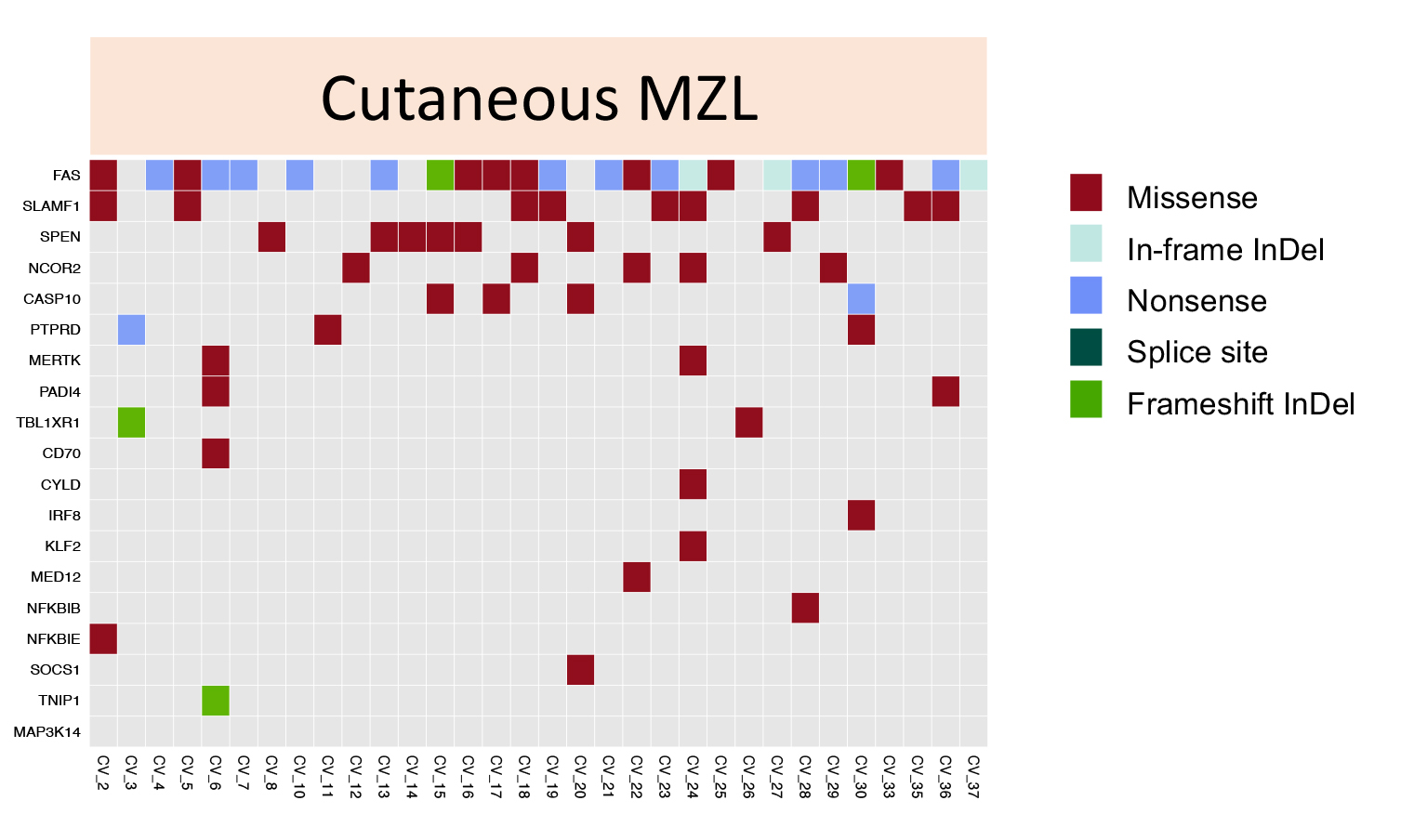

Supplement: Supplementary file 12 — (JPG 321 kb) [file 428_2021_3186_MOESM12_ESM.jpg]

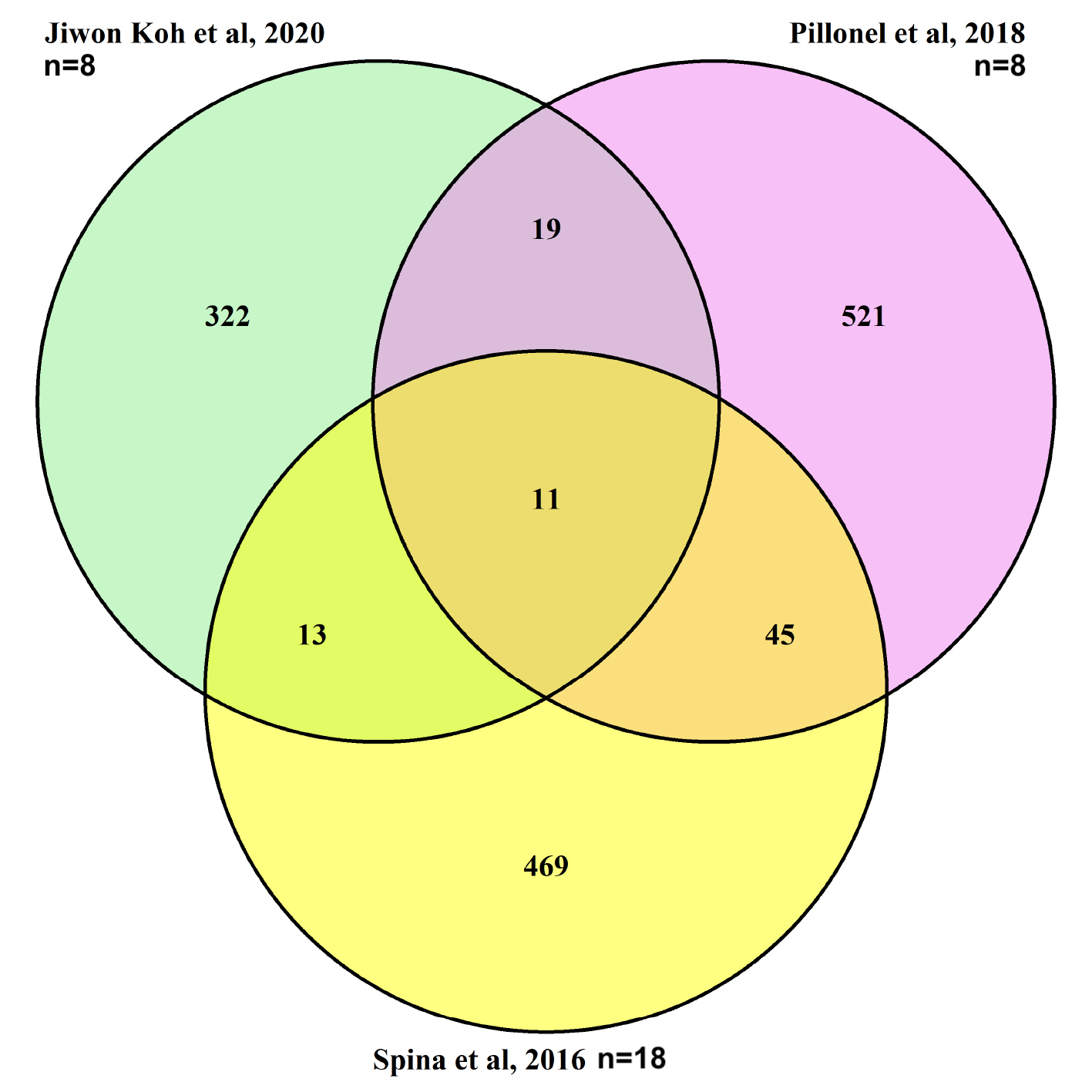

Supplement: Supplementary file 13 — (JPG 332 kb) [file 428_2021_3186_MOESM13_ESM.jpg]
